# Expression of Na^+^/K^+^-ATPase Was Affected by Salinity Change in Pacific abalone *Haliotis discus hannai*

**DOI:** 10.3389/fphys.2018.01244

**Published:** 2018-09-07

**Authors:** Yanglei Jia, Xiao Liu

**Affiliations:** ^1^Key Laboratory of Experimental Marine Biology, Institute of Oceanology and Center for Ocean Mega-Science, Chinese Academy of Sciences, Qingdao, China; ^2^Laboratory for Marine Biology and Biotechnology, Qingdao National Laboratory for Marine Science and Technology, Qingdao, China; ^3^University of Chinese Academy of Sciences, Beijing, China

**Keywords:** salinity, osmoregulation, ion transportation, Na^+^/K^+^-ATPase, cAMP

## Abstract

Na^+^/K^+^-ATPase (NKA) belongs to the P-type ATPase family, whose members are located in the cell membrane and are distributed in diverse tissues and cells. The main function of the NKA is to regulate osmotic pressure. To better understand the role of NKA in osmoregulation, we first cloned and characterized the full-length cDNAs of NKA α subunit and β subunit from Pacific abalone *Haliotis discus hannai* in the current study. The predicted protein sequence of the NKA α subunit, as the catalytic subunit, was well conserved. In contrast, the protein sequence of the β subunit had low similarity with those of other species. Phylogenetic analysis revealed that both the α and β subunits of the NKA protein of Pacific abalone were clustered with those of the Gastropoda. Then, the relationship between salinity changes and the NKA was investigated. Sudden salinity changes (with low-salinity seawater (LSW) or high-salinity seawater (HSW)) led to clear changes in ion concentration (Na^+^ and K^+^) in hemolymph; however, the relative stability of ion concentrations in tissue revealed that Pacific abalone has a strong osmotic pressure regulation ability when faced with these salinity changes. Meanwhile, the expression and activity of the NKA was significantly decreased (in LSW group) or increased (in HSW group) during the ion concentration re-establishing stages, which was consistent with the coordinated regulation of ion concentration in hemolymph. Moreover, a positive correlation between cyclic adenosine monophosphate (cAMP) concentrations and NKA mRNA expression (NKA activity) was observed in mantle and gill. Therefore, the sudden salinity changes may affect NKA transcription activation, translation and enzyme activity via a cAMP-mediated pathway.

## Introduction

Abalones are large marine snails in the family Haliotidae and the genus Haliotis belonging to the class Gastropoda of the phylum Mollusca (Lv, 1978; Appeltans et al., 2014; Cook, 2016). Pacific abalone *Haliotis discus hannai*, which is naturally distributed in the Northwest Pacific, is considered an important commercially fishery industry animal in China. The production in 2015 was about 115,397 metric tons, accounting for approximately 90% of the worldwide yield (Elliott, 2000; Gordon and Cook, 2004; Cook, 2016).

Temperature and salinity are the primary physical factors affecting the distribution and physiological metabolism of shellfish (Davenport et al., 1975; Burton, 1983; Navarro, 1988). Pacific abalone is a stenohaline species, and it is very sensitive to low salinity. The strain of P-97, which had experienced several generations of linearly selection since 1997, resulting in a significantly increased tolerance of temperature and salinity. The optimum growth salinity for the sixth generation of P-97 is approximately 24–36 part per thousand (ppt) (Kong et al., 2017).

The main factors related to osmotic pressure are H_2_O and the concentrations of ions and bioorganic molecules. A cell’s osmolarity is the sum of the concentrations of the various ion species and many proteins and other organic compounds inside the cell. The main ions include Na^+^, K^+^, Ca^2+^, and Cl^-^, and bioorganic molecules include free amino acids (Siebers et al., 1972; Amende and Pierce, 1980). Na^+^ and Cl^-^ are the principal osmotically active solutes in the hemolymph. Contribution of Na^+^ and Cl^-^ accounted for 47.7–51.6% and 37.1–42.2% of the total osmotic concentration of the hemolymph, respectively (Cheng et al., 2002). The combined contribution of Na^+^ and Cl^-^ to the hemolymph osmolality is about 90%. It is suggested that the contribution of organic compounds like protein, free amino acid to the osmotic concentration of the hemolymph is much lower than that of ions. The movements of most ions through the membrane are mediated by membrane transport proteins which are specialized to varying degrees in the transport of specific ions. The transmembrane transport of Na^+^ and Cl^-^ could be mediated by several channel proteins like sodium channels, some members of electroneutral cation-Cl cotransporters (solute carrier family, SLC12A) and many others. Na^+^/K^+^-ATPase (NKA) is one of the ion channels that using the energy derived from ATP hydrolysis to maintain the membrane potential by driving three sodium ions export and two potassium ions import across the plasma membrane against their electrochemical gradients. There is hence a net export of a single positive charge per pump cycle. In most animal cells, the NKA is responsible for about 1/5 of the cell’s energy expenditure. The NKA is an integral membrane heterodimer belonging to the P-type ATPase family and it is an enzyme that found in the membranes of all animal cells (Hall and Guyton, 2006; Clausen et al., 2017). It is composed of two catalytic α subunits and two non-catalytic β subunits (Therien and Blostein, 2000). Although the β subunit has no catalytic activity, it is required for stabilization, maturation, and translocation of the catalytic α subunit to the plasma membrane (Beggah et al., 1997; Hasler et al., 1998; Rajasekaran et al., 2004). In addition, the β subunit also mediates the trans-dimerization of NKAs between neighboring cells, where they regulate the integrity of tight junctions (Rajasekaran and Rajasekaran, 2003; Babdinitz et al., 2009; Tokhtaeva et al., 2011). Furthermore, a third, non-essential subunit (the γ subunit) has been identified in mammals (Reeves et al., 1980). Thus far, four α, three β and one γ subunit isoforms have been identified in mammals, and several α and β subunits have been identified in fish (Mobasheri et al., 2000; Guynn et al., 2002).

Previous studies have determined that the influence of salinity on NKAs in many crustacean and vertebrates, such as fish, and NKA is highly sensitive to the changes in salinity (Mancera and McCormick, 2000; Richards et al., 2003; Pan et al., 2014; Pham et al., 2016). However, the responses of NKA were not consistently when faced with the changes of salinity in different species and the detailed mechanism is not clear yet. In *Litopenaeus vannamei*, NKA α-subunit gene transcript increased rapidly when the salinity decreased (Pan et al., 2014). Whereas in *Fundulus heteroclitus*, the increased salinity induced a rise in gill NKA activity 3 h after transfer (Mancera and McCormick, 2000). In *Oncorhynchus Mykiss*, five NKA α-isoforms were identified. High salinity stimulation decreased NKA α1a mRNA, but increased NKA α1b mRNA. The other 3 isoforms were not changed following salinity change (Richards et al., 2003). Moreover, there has been less research on NKA in mollusk. Many studies have confirmed that salinity change could stimulate the change in nervous endocrine (like dopamine (DA), noradrenaline (NE) and 5-hydroxytryptamine (5-HT)), thereby inducing the change in physiological function (Sommer and Mantel, 1991; Hoshijima and Hirose, 2007). Morris and Edwards (1995) also confirmed that the injection of DA or cyclic adenosine monophosphate (cAMP) have the same effect on NKA activity. These researches indicated that biogenic amines play a role in stimulating the NKA.

The action of most biogenic amines combine with the G protein-coupled receptor (GPCR) and is then mediated by the generation of intracellular cAMP, a very important second messenger in many biological processes. The cAMP molecule is a derivative of ATP and used for intracellular signal transduction in many different organisms, conveying the cAMP-dependent pathway (Bos, 2006). The predominant effect of cAMP is to activate a cAMP-dependent protein kinase (PKA). Four molecules of cAMP bind each dormant PKA holoenzyme, activating the kinase by releasing the catalytic subunits from a regulatory subunit-cAMP complex. Then, the catalytic subunit of PKA could get into the nucleus and subsequently regulate gene transcription. The detailed mechanisms between cAMP and NKA activity have been well studied in vertebrates like rats. Previous literatures reported that the enzyme activity of NKA is thought to be downregulated by cAMP via PKA-dependent phosphorylation of the catalytic subunit (Bertorello et al., 1991; Kurihara et al., 2000). Whereas in another study, PKA-mediated stimulation of NKA α subunit phosphorylation level likely participates in the cAMP-dependent stimulation of NKA activity (Kiroytcheva et al., 1999). Whether a salinity change could affect the content of cAMP and then regulate the gene expression and activity of NKA in Pacific abalone needs further research.

According to the literatures mentioned above, we could conclude that NKA may play an important role in salinity regulation and the responses of NKA were not consistent between different species. Moreover, the effect of salinity on NKA has not been reported in Pacific abalone. The present study described the molecular cloning and characterization of full-length NKA (α subunit and β subunit) cDNA from Pacific abalone (designated HdNKA) as well as its expression profile in various tissues. In addition, we then determined the influence of salinity on the expression and activity of HdNKA. Furthermore, we measured the concentration of ions and cAMP in both hemolymph and tissues. The results were used to elucidate the correlation between cAMP and HdNKA (expression and activity) in Pacific abalone *Haliotis discus hannai*.

## Materials and Methods

### Animal

Adult abalones (length: 40 ± 5 mm, weight: 4.8 ± 0.3 g) that derived from a selective breeding population known as P-97, which had experienced seven generations of selection since 1997 was obtained from a commercial farm in Xunshan, Weihai, China. After transported to the laboratory, animals were adapted for 1 week in tank filled with aerated normal seawater (NSW, salinity = 30.0 ppt) at 24.0 ± 0.5°C. Low salinity seawater (LSW, salinity = 20.0 ppt) was a blend of fresh water and NSW. High salinity seawater (HSW, salinity = 40.0 ppt) was a blend of NSW and marine life reef salt (Goe, Qingdao, China). Salinity was measured by water quality handheld instrument YSI pro30 (YSI, Yellow Springs, OH, United States). During the experimental period, the Pacific abalones were fed with a formulated commercial diet daily (Gold Feed, Weihai, China).

### Experimental Design

**FIGURE 1:**
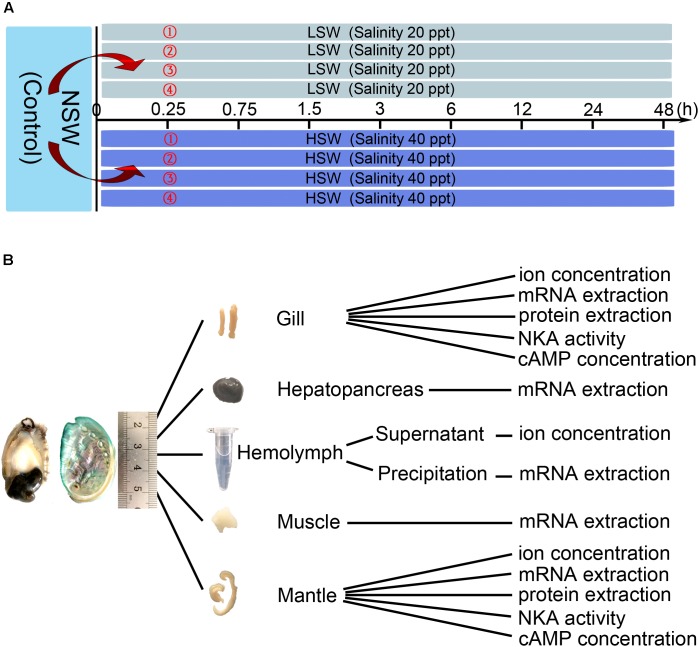
Experimental design. **(A)** Treatment and sampling time points. **(B)** Application of sampled tissue.

As shown in **Figure 1**, Pacific abalones were transferred directly into four tanks filled with LSW or HSW (80 individuals per group, 20 individuals per tank). After the sudden transfer (all LSW and HSW treatment groups), animals were sampled at 0.25, 0.75, 1.5, 3, 6, 12, 24, and 48 h (8 individuals per point, 2 individuals per tank). Pacific abalones grown in NSW were sampled at 0 h, and these samples were regarded as the control. Pacific abalone was euthanized as previously described (Altenhein et al., 2002). The hemolymph (∼800 μl) was withdrawn from the sole side foot muscle and collected into 1.5 ml Eppendorf (EP) tubes carefully. Then, the hemolymph was centrifuged at 1000 rpm for 10 min (Heraeus Pico 21, 24 × 1.5/2.0 ml rotor, Thermo Scientific, Carlsbad, CA, United States). Supernatants were separated into a new tube and then stored at -80°C. Different tissues, including hepatopancreas, muscle, mantle and gill, were dissected. Tissues were washed with deionized water rapidly, wiped with filter paper and then evenly separated into several parts for the determination of ion concentration, cAMP concentration, and *Hdnka* expression and activity. The tissue was immersed into liquid nitrogen for quick freezing and then stored at -80°C. All animal experiments were performed with the approval of the Institutional Animal Ethics Committee of Institute of Oceanology, Chinese Academy of Sciences.

### Preparation of Total RNA for the First Stranded Synthesis

Total RNA of each tissue was extracted with TRIzol Reagent (Cat No. 15596-026, Invitrogen, Carlsbad, CA, United States) according to the manufacturer’s instructions. The concentration of RNA was determined by measuring the OD at 260 nm with the NanoDrop ND-1000 UV-Visible Spectrophotometer (Thermo Scientific, Carlsbad, MA, United States). The integrity was checked by separating the RNA on a 1% agarose gel and staining with Gel-Red (Cat No. D0139, Beyotime, Shanghai, China).

### Cloning of the Full Length cDNA of Na^+^/K^+^-ATPase α Subunit and β Subunit

Total RNA extracted from different tissue at 0 h point was mixed and used for 5′ RACE and 3′ rapid-amplification of cDNA ends (RACE). First-strand cDNA were synthesized using SMARTer^®^ RACE 5′/3′ Kit (Cat No. 634858, TAKARA, Dalian, China) following the manufacturer’s instructions. On the basis of the fragments of NKA α subunit and β subunit gene obtained from transcriptome sequencing (unpublished data), the primers used for amplification of the full length of NKA α subunit and β subunit were designed using Primer Premier 5 (PREMIER Biosoft International, Palo Alto, CA, United States) following the kit protocol and synthesized by Beijing Genomics Institute (BGI) (**Table 1**). The PCR reaction was conducted in the following conditions: 5 cycles of denaturation (94°C for 30 s), annealing and elongation (72°C for 3 min); then 5 cycles of denaturation (94°C for 30 s), annealing (70°C for 30 s) and elongation (72°C for 3 min); finally, 25 cycles of denaturation (94°C for 30 s), annealing (68°C for 30 s) and elongation (72°C for 3 min). A final extension step was conducted at 72°C for 10 min. The PCR products were electrophoresed on a 1% agarose gel and staining with Gel-Red. Those bands with the expected sizes were extracted using NucleoSpin Gel and PCR Clean-Up Kit (Cat No. 740609.10, MACHEREY-NAGEL, Düren, Germany). The purified PCR products were then subcloned using In-Fusion HD Cloning Kit (Cat No. 638909, TAKARA, Dalian, China) and sequenced (Sanger dideoxy sequencing) in both directions.

**Table 1:** List of primer sequences used in this study.

Primer name	Primer sequence (5′-3′)	Amplification target
NKAα-5′	***GATTACGCCAAGCTT*** CACTGGGTGTGACAGCATAGCGGTAG	For NKA α subunit 5′ RACE
NKAα-3′	***GATTACGCCAAGCTT*** CAGTTCAGCCCCATCACACTCTCCC	For NKA α subunit 3′ RACE
NKAβ-5′	***GATTACGCCAAGCTT*** CATAGCCTTGCTTTGGCAGGTACTCG	For NKA β subunit 5′ RACE
NKAβ-3′	***GATTACGCCAAGCTT*** TTCACCATTTGTCAGCACATTCCCA	For NKA β subunit 3′ RACE
UPM	CTAATACGACTCACTATAGGGCAAGCAGTGGTATCAACGCAGAGT	Universal Primer for RACE PCR
qactb-F	GGTATCCTCACCCTCAAGT	For actb q-PCR
qactb-R	GGGTCATCTTTTCACGGTTG	
qNKAα-F	TACATCCACCATCTGCTCG	For NKA α subunit q-PCR
qNKAα-R	AAGTTTGAATTCGGCCC	
qNKAβ-F	TAATGAGAACCTGGGTGAGA	For NKA β subunit q-PCR
qNKAβ-R	GATAAATACAAGGGGCGAC	

*Letters in bold and italics indicate the nucleobase added to the 5′-end of GSP that used for In-Fusion cloning*.

### Bioinformatics and Phylogenetic Analysis

The sequences were assembled using DNAMAN 9 (Lynnon Biosoft, San Ramon, CA, United States). Then the open reading frame (ORF) was analyzed. Amino acid sequences were aligned using the ClustalX2 (Larkin et al., 2007), and phylogenetic tree was carried out in MEGA 7.0 program using maximum likelihood (ML) method (Kumar et al., 2016). Bootstrap value was computed over 1000 replications. The similarity between different sequences was calculated by DNAMAN 9 software. The trans-membrane domains of protein sequence were predicted by the TMHMM Server v. 2.0^1^ and MEMSAT3 and MEMSAT-SVM (Buchan et al., 2010). Sequences and accession numbers were shown in Supplementary Files.

### Physiological and Biochemical Indexes Measurement

The frozen hemolymph was thawed on ice and recentrifuged at 5000 rpm for 10 min. The supernatant was separated into a new EP tube for hemolymph ion concentration measurements. Approximately 50 mg tissue was put in a glass homogenizer with 200 μl ice-cold deionized water and homogenized completely. Then, it was centrifuged at 5000 rpm for 10 min. The supernatant was transferred to a new EP tube. The supernatants were used for tissue ion concentration measurements. The tissues used for NKA activity were homogenized with normal saline (0.9%) and centrifuged at 5000 rpm for 10 min for collection of the supernatant. The tissue used to determine the cAMP concentration was homogenized with 0.1 M HCl and centrifuged at 13000 rpm for 10 min for collection of the supernatant. All of the homogenate steps were carried out on ice, and the centrifugal steps were carried out at 4°C. The supernatants were separated into new tubes and stored at -80°C. The protein concentrations of supernatants after tissue homogenization were measured with a Protein Quantitative Reagent Kit-BCA Method (Cat No. P0010, Beyotime, Shanghai, China).

The concentration of Na^+^ ions was determined using a Blood Sodium Concentration Assay Kit (Cat No. BC2805, Solarbio, Beijing, China). The concentration of K^+^ ions was determined using a Potassium Assay Kit according to the manufacturer’s instructions (Cat No. C001-2, Nanjing Jiancheng Bioengineering Institute, Nanjing, China). The activity of NKA was measured using a Na^+^/K^+^-ATPase assay kit (Cat No. A070-2, Nanjing Jiancheng Bioengineering Institute, Nanjing, China). The concentration of cAMP in tissue was measured with a cAMP Direct Immunoassay Kit (Colorimetric) (Cat No. K371-100, BioVision, Milpitas, CA, United States).

### Q-PCR Analysis of Na^+^/K^+^-ATPase α Subunit and β Subunit

Q-PCR was used to determine the influence of salinity on the expression of NKA α subunit and β subunit. The first-strand cDNA used for q-PCR was synthesized using PrimeScript^TM^ RT reagent Kit with gDNA Eraser (Cat No. RR047A, TAKARA, Dalian, China). SYBR^®^ Premix Ex^TM^ Taq II kit (Cat No. RR42LR, TAKARA, Dalian, China) was used to perform the PCR reactions. The primers designed using Primer Premier 5 following the manufacturer’s instructions and synthesized by BGI (**Table 1**). The following PCR program was used: 95°C for 30 s, followed by 40 cycles of 95°C for 5 s, 60°C for 30 s. Melting curve was inserted to analyze the specific of PCR products. The q-PCR reaction was performed using the QuantStudio^TM^ 6 Flex Real-Time PCR System (Applied Biosystems, Carlsbad, CA, United States). The amplicon sizes and amplification efficiency of primers used for q-PCR were shown in **Supplementary Figure S1**. In addition, the specificity of the primers that we used was measured (**Supplementary Figure S1**). The data were calculated with the 2^-ΔΔC_t_^ method. Beta-actin (actb, Accession number: AY380809.1) was used as the internal control and the initial group (0 h) was used as the normalized group.

### Western Blotting Analysis of Na^+^/K^+^-ATPase α Subunit

The membrane proteins of each tissue were extracted with RIPA buffer (high) (Cat No. R0010, Solarbio, Beijing, China). The concentration of protein was measured with a Protein Quantitative Reagent Kit-BCA Method. Protein samples (extracted from mantle or gill at the same time point) were mixed depending on their concentration and then subjected to SDS-PAGE; the separated proteins were then electrophoretically transferred onto a PVDF membrane. In brief, the membrane was blocked with 5% skim milk in TBST at room temperature for 1 h. Then, the blots were incubated with primary antibodies against the NKA α subunit (1:500, ATP1A2 Rabbit Polyclonal, Cat. No. 16836-1-AP, Proteintech, Rosemont, IL, United States) overnight at 4°C, followed by incubation with appropriate peroxidase-conjugated secondary antibodies (1:3000, HRP Goat Anti-Rabbit IgG (H+L), Cat. No. AS014, ABclonal, Wuhan, China). Gray values were analyzed using ImageJ software. Actb served as an internal control (1:1000, ACTB Monoclonal Antibody, Cat. No. AC026, ABclonal, Wuhan, China), and the initial group (0 h) was used as the normalized group. The detail information and the specificity of primary antibody against HdNKA α and actb were shown in **Supplementary Figure S1**.

### Statistical Analysis

Statistical analysis was performed with the Statistical Package for the Social Sciences, version 18.0 (SPSS Inc., Chicago, IL, United States). The results are presented as the means ± SEM, and differences were considered significant at the *P* < 0.05 level. The correlations between ion concentrations and salinity were analyzed using linear regression. Differences in all time-points and salinity groups were determined using two-way analysis of variance followed by multiple comparison tests. Since the data did not pass the Shapiro–Wilk normality test, Spearman’s test was carried out to determine the correlation between cAMP concentration and *Hdnka* mRNA expression and HdNKA activity in mantle and gill.

## Results

### Ion Concentration

**FIGURE 2:**
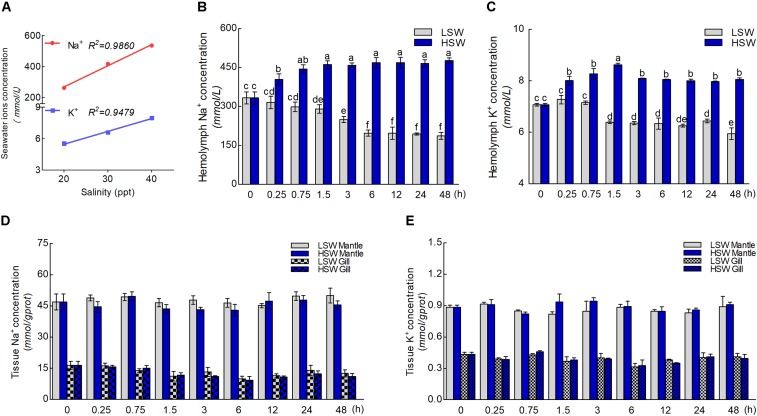
Concentrations of Na^+^ and K^+^ ions. **(A)** The correlation between salinity and concentrations of Na^+^ and K^+^. **(B)** The concentration of Na^+^ in hemolymph after the sudden salinity change at different times. **(C)** The concentration of K^+^ in hemolymph after the sudden salinity change at different times. **(D)** The concentration of Na^+^ in mantle and gill after the sudden salinity change at different times. **(E)** The concentration of K^+^ in mantle and gill after the sudden salinity change at different times. Different letters on data indicate statistically significant differences between the results (*P* < 0.05). *n* = 4/time-point.

The concentration of Na^+^ and K^+^ in LSW, HSW, and NSW were measured in order to test the differences in aquaculture waters with different salinities (**Figure 2A**). These data indicated that the ion concentration and salinity of seawater are positively correlated (*P <* 0.0001). A higher salinity represented a higher concentration of Na^+^ and K^+^. To gain more insight into the influence of salinity on the ionic concentration in hemolymph, we measured the concentrations of Na^+^ and K^+^ in hemolymph after the sudden treatment with LSW or HSW at different times (**Figures 2B,C**). From these data, we found that the ion concentrations of Na^+^ and K^+^ in samples treated with LSW gradually decreased as the treatment time increased. In contrast, the HSW treatment produced a significant increase in both Na^+^ and K^+^ concentrations (*P* < 0.05). In addition, the concentrations of Na^+^ and K^+^ in mantle and gill after the treatment at different times were measured (**Figures 2D,E**). These data showed that the trend in Na^+^ and K^+^ concentrations in the mantle and gill fluctuated, but no significant differences were observed between different groups after treatments with LSW and HSW (*P* > 0.05).

### Sequence Analysis of Na^+^/K^+^-ATPase α Subunit and β Subunit

**FIGURE 3:**
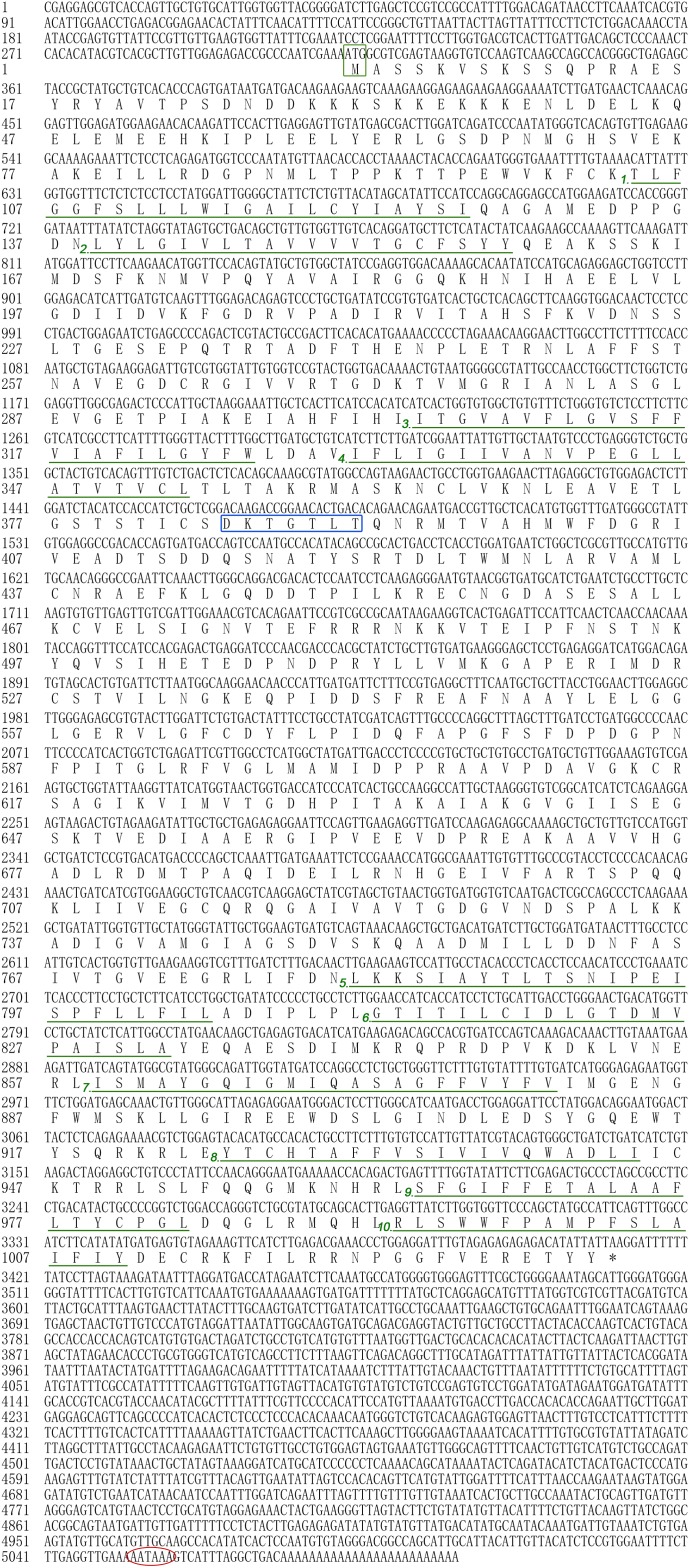
The cDNA sequence and deduced amino acid sequence of the Na^+^/K^+^-ATPase α subunit from *H. discus hannai* (Accession number: MG767304). The start codon (ATG) is boxed in green. The asterisk (^∗^) indicates the stop codon. The P-type ATPase motif is indicated by a blue box. A polyadenylation signal sequence AATAAA is circled with a red oval. The transmembrane regions are underlined with green and numbered (1–10).

**FIGURE 4:**
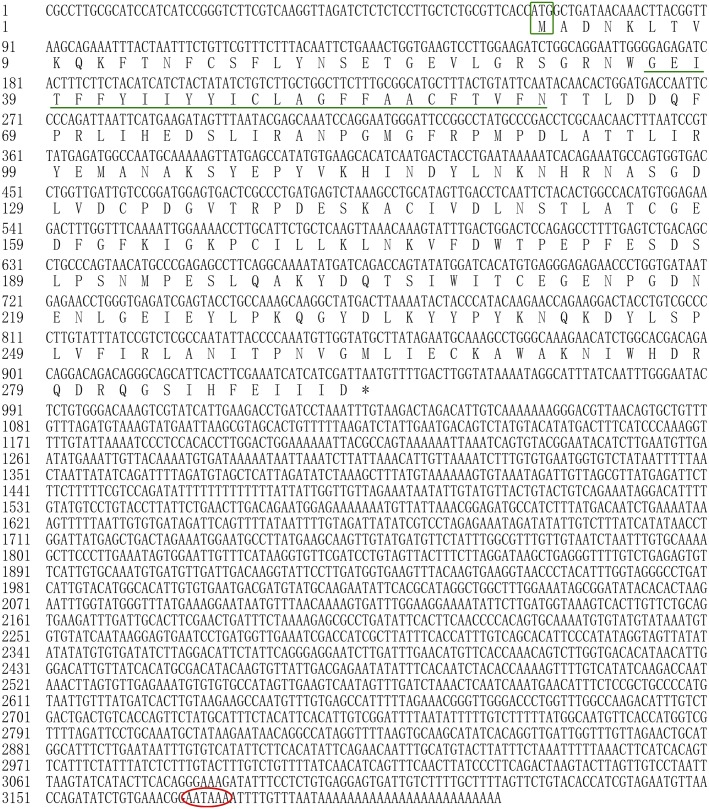
The cDNA sequence and deduced amino acid sequence of the Na^+^/K^+^-ATPase β subunit from *H. discus hannai* (Accession number: MG767305). The start codon (ATG) is boxed in green. The asterisk (^∗^) indicates the stop codon. A polyadenylation signal sequence AATAAA is circled with a red oval. The transmembrane region is indicated with green underline.

In the present study, all of the *Hdnka* α subunit and β subunit cDNA sequences were identified by RACE-PCR from the Pacific abalone. The nucleotides and deduced amino acid sequences are shown in **Figures 3**, **4**. The full-length cDNA of the *Hdnka* α subunit was 5098 bp long and contained a 5′ UTR of 312 bp, a 3′ UTR of 1690 bp with a poly (A) tail, and an ORF of 3096 bp encoding a polypeptide of 1032 amino acids (GenBank accession number: MG767304, **Figure 3**). The full-length cDNA of the *Hdnka* β subunit was 3213 bp long and contained a 5′ UTR of 65 bp, a 3′ UTR of 2272 bp with a poly (A) tail, and an ORF of 876 bp encoding a polypeptide of 292 amino acids (GenBank accession number: MG767305, **Figure 4**).

**FIGURE 5:**
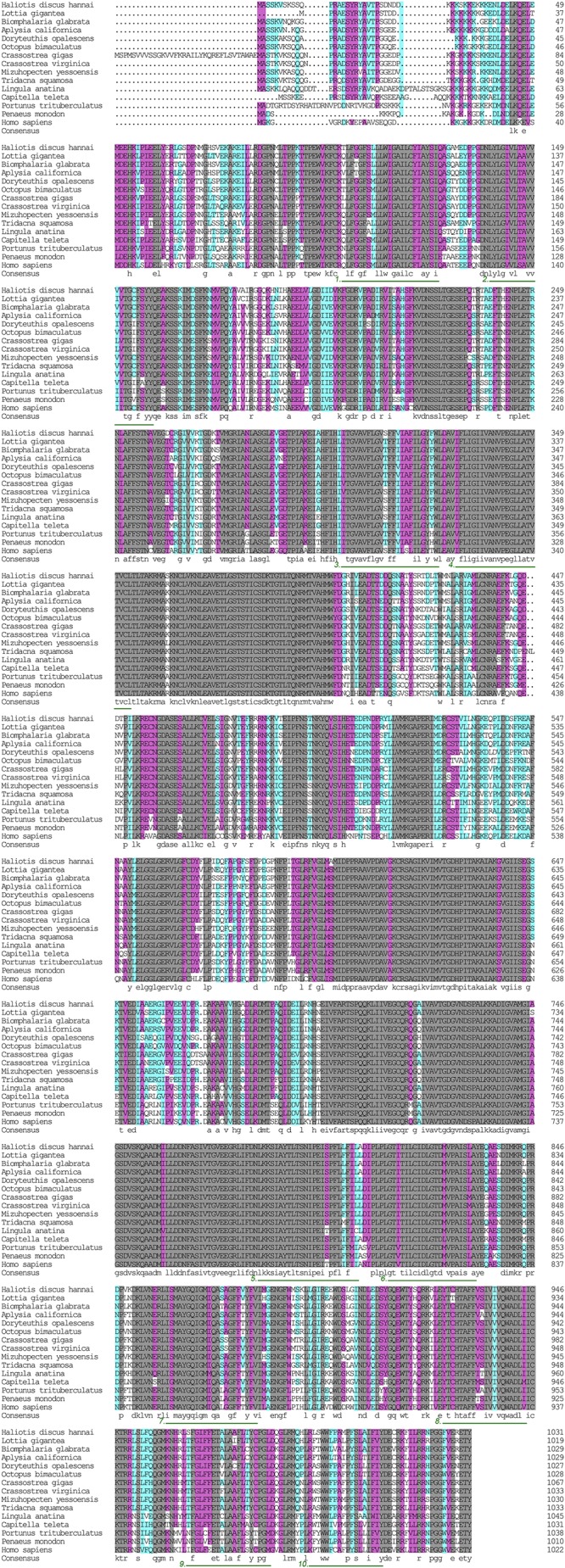
Multiple alignment of the HdNKA α subunit sequence with NKA α subunit sequences deposited in GenBank. The amino acids highlighted in gray are identical in all sequences. The amino acids highlighted in pink are conserved more than 75%. The amino acids highlighted in cyan are conserved more than 50%. The regions underlined with green and numbered (1–10) are transmembrane regions.

**FIGURE 6:**
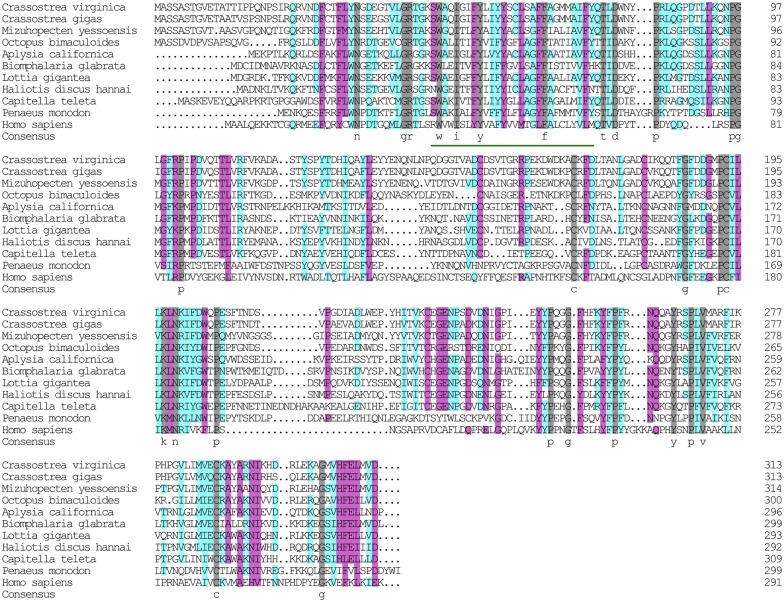
Multiple alignment of the HdNKA β subunit sequences with NKA β subunit sequences deposited in GenBank. The amino acids highlighted in gray are identical in all sequences. The amino acids highlighted in pink are conserved more than 75%. The amino acids highlighted in cyan are conserved more than 50%.

**FIGURE 7:**
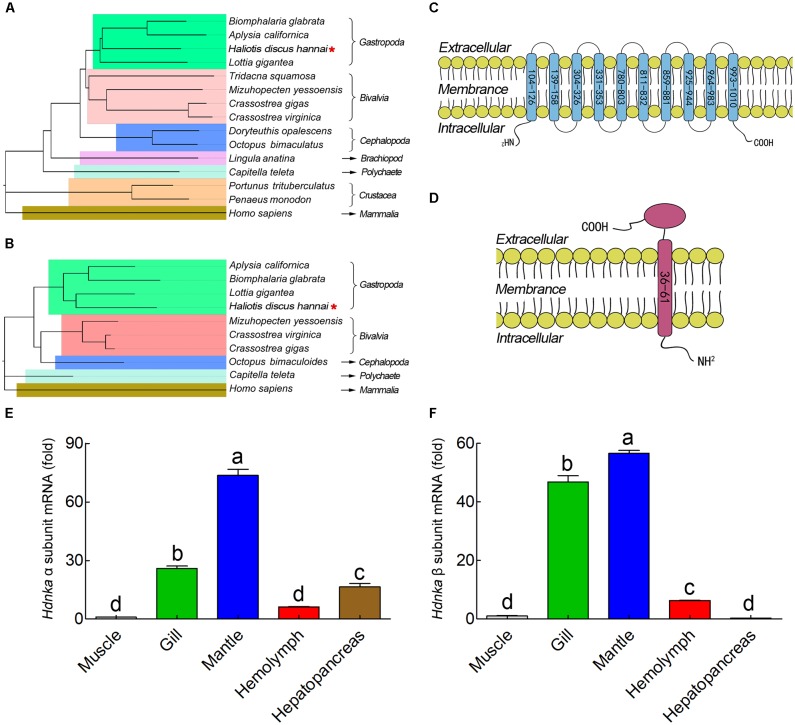
Phylogenetic tree, transmembrane structure and expression profile analysis. **(A)** Phylogenetic tree analysis of HdNKA α subunit from Pacific abalone and NKA α subunits from other species. **(B)** Phylogenetic tree analysis of HdNKA β subunit from Pacific abalone and NKA β subunits from other species. **(C)** Topology model of the α subunit of HdNKA. The yellow spheroids represent the cell membrane, and the blue rod-like structure represents the transmembrane region. The numbers in the rod represent the amino acid sites. **(D)** Topology model of the HdNKA β subunit. The yellow spheroids represent the cell membrane. The red rod and oval structures represent the transmembrane region and highly glycosylated extracellular domain, respectively. The numbers in the rod represent the amino acid sites. **(E)** Transcript levels of *Hdnka* α subunit in the tissues obtained from Pacific abalone. **(F)** Transcript levels of *Hdnka* β subunit in the tissues obtained from Pacific abalone. Different letters on data indicate statistically significant differences between the results (*P* < 0.05). *n* = 4/time-point.

The α subunit of NKA is the major catalytic unit. Multiple sequence alignments showed that the amino acid sequence of the *Hdnka* α subunit shared high similarity with those of Gastropoda (87.31–89.24%), Bivalvia (85.01–87.31%), Cephalopoda (85.27–85.76%) and *Homo sapiens* (73.55%). Transmembrane analysis showed that the high similarity regions of the sequence are the transmembrane regions (**Figure 5**). However, the multiple sequence alignments for the *Hdnka* β subunit amino acid sequences showed low similarity when compared with those of other species [Gastropoda (43.05–47.46%), Bivalvia (40.38–44.13%), Cephalopoda (40.85%) and *Homo sapiens* (23.96%)] (**Figure 6**). Phylogenetic tree analyses revealed that both the α subunit and β subunit of NKA in Pacific abalone clustered with those from other invertebrate species (especially those characterized in other marine Gastropoda and Bivalvia) (**Figures 7A,B**).

In terms of structure, TMHMM and MEMSAT3 and MEMSAT-SVM analysis showed that the *Hdnka* α subunit is a transmembrane protein. Transmembrane protein topology prediction showed that the transmembrane region possesses 10 transmembrane helices. Both the C-terminus and the N-terminus were localized on the intracellular side (**Figure 7C**). The β subunit of *Hdnka* is characterized by an intracellular N-terminus, followed by only one α-helix, and an extracellular C-terminus (**Figure 7D**). Furthermore, the extracellular domain was highly glycosylated (Nyblom et al., 2013).

### Gene Expression of Na^+^/K^+^-ATPase α Subunit and β Subunit

The expression of the *Hdnka*α and β subunits occurred in all tissues examined (**Figures 7E,F**), including muscle, gill, mantle, hemolymph and hepatopancreas in NSW group. Muscle was used as the normalized group. The highest expression levels of both the *Hdnka* α and β subunits were found in mantle. The lowest expression level of the *Hdnka* α subunit was found in muscle, while that of the β subunit was found in hepatopancreas.

**FIGURE 8:**
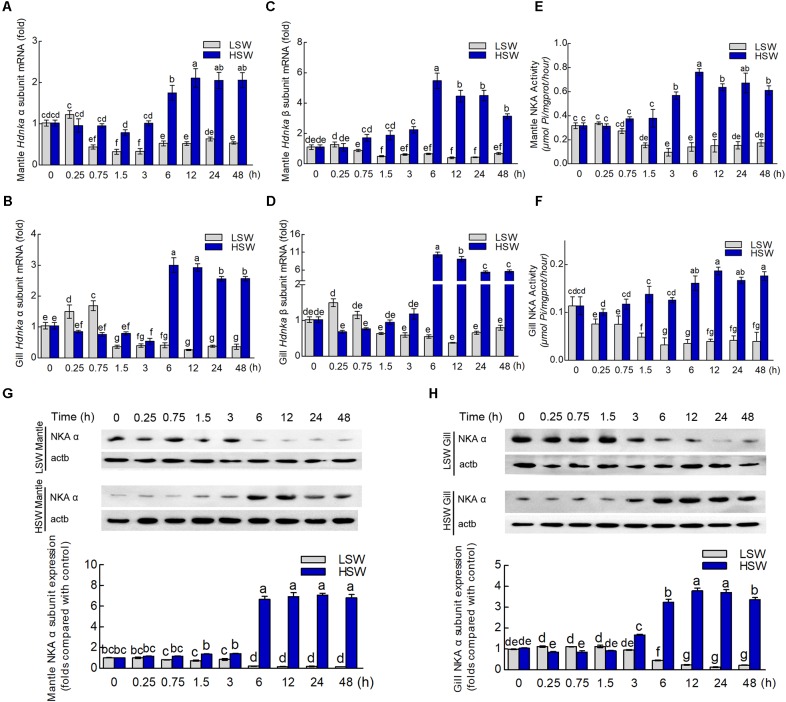
Expression and activity analysis of Na^+^/K^+^-ATPase α and β subunits. **(A)** Influence of salinity changes on the mRNA level of the *Hdnka* α subunit in mantle. **(B)** Influence of salinity changes on the mRNA level of the *Hdnka* β subunit in mantle. **(C)** Influence of salinity changes on the mRNA level of the *Hdnka* α subunit in gill. **(D)** Influence of salinity changes on the mRNA level of the *Hdnka* β subunit in gill. **(E)** Influence of salinity changes on the activity of HdNKA in mantle. **(F)** Influence of salinity changes on the activity of HdNKA in gill. **(G)** Influence of salinity changes on the expression of the HdNKA α subunit in mantle determined with western blot. **(H)** Influence of salinity changes on the expression of the HdNKA α subunit in gill determined with western blot. Different letters on data indicate statistically significant differences between the results (*P* < 0.05). *n* = 4/time-point.

To evaluate the influence of salinity changes on *Hdnka* expression, we examined the mRNA levels of both the *Hdnka* α and β subunits in mantle and gill after sudden treatment with LSW or HSW at different times. The mRNA expression levels of the *Hdnka* α subunit increased slightly at the initial time, after the treatment of LSW, in mantle (*P* > 0.05) and gill (*P* < 0.05). However, as the time of treatment increased, the expression decreased. After treatment for approximately 1.5 h, the expression was reduced to its minimum. Then, the expression was slightly higher in mantle, but it was still significantly lower than the normal level (*P* < 0.05) (**Figures 8A,C**). In contrast, treatment with HSW first decreased the expression of the *Hdnka* α subunit slightly in gill (*P* < 0.05). However, the reduction in mantle was not significant (*P* > 0.05). After treatment for 6 h, there was a significant increase in this expression in both these two tissues. This high expression pattern continued in the subsequent processing (*P* < 0.05). The influence of salinity on the mRNA level of the *Hdnka* β subunit was similar to its influence on the expression of the α subunit (**Figures 8B,D**).

### Na^+^/K^+^-ATPase Activity

To further investigate whether the activity of HdNKA is under osmotic regulation, we carried out the measurement of HdNKA activity after the sudden treatment, including measuring in the tissues of mantle and gill (**Figures 8E,F**). From these data, we could find that at the initial period of the experiment, the activity of HdNKA was very stable. There was no difference between them (*P* > 0.05). However, as time progressed, a dramatic change occurred in the HdNKA activity. The LSW treatment led to a rapid reduction in expression in the tissues of both mantle and gill. In contrast, the activity of HdNKA was significantly increased in these tissues after the HSW treatment. These data indicated that the salinity had a significant influence on HdNKA activity in both mantle and gill.

### Western Blotting Analysis

We used western blotting to determine whether the salinity change stimulation could affect the protein level of HdNKA. The amount of HdNKA α subunit protein in mantle and gill was examined after the LSW and HSW treatments and further measured by densitometry (**Figures 8G,H**). A highly significant reduction in HdNKA α subunit expression was found in the LSW treatment group relative to its level in the control. In contrast, the HSW treatment led an obvious increase in the expression of HdNKA α subunit. These data were consistent with the q-PCR results.

### cAMP Concentration

**FIGURE 9:**
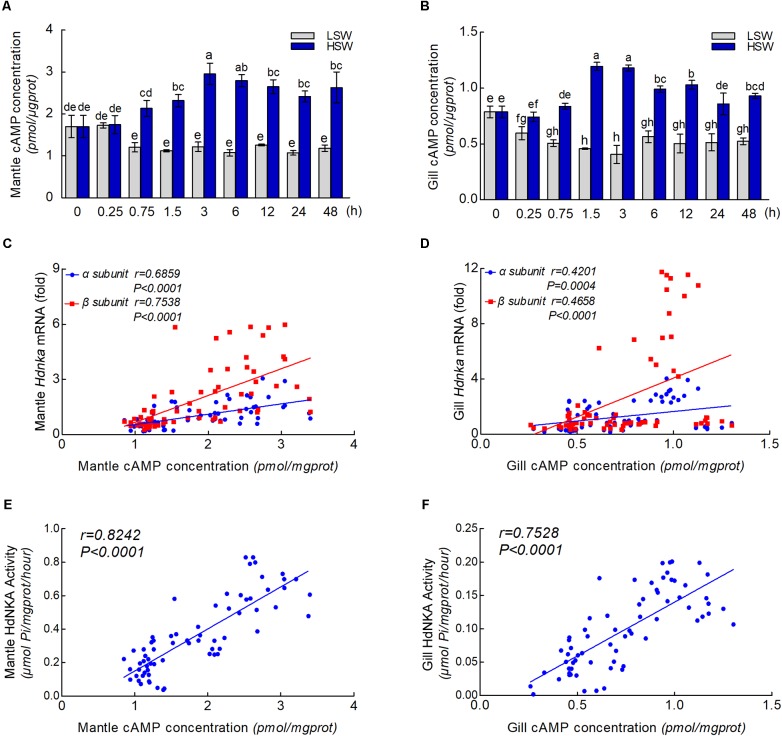
cAMP concentrations and correlation analysis. **(A)** Influence of salinity changes on the concentration of cAMP in mantle. **(B)** Influence of salinity changes on the concentration of cAMP in gill. **(C)** Correlation analysis between cAMP concentration and *Hdnka* α and β subunit expression in mantle. **(D)** Correlation analysis between cAMP concentration and *Hdnka* α and β subunit expression in gill. **(E)** Correlation analysis between cAMP concentration and HdNKA activity in mantle. **(F)** Correlation analysis between cAMP concentration and HdNKA activity in gill. Different letters on data indicate statistically significant differences between the results (*P* < 0.05). *n* = 4/time-point.

To further explore the underlying molecular mechanism by which osmotic regulation leads to significant changes in HdNKA expression and activity after sudden treatment with different salinities, we examined the concentration of cAMP in both mantle and gill (**Figures 9A,B**). We found that the difference in cAMP concentration was statistically significant after the different salinity treatments at different times (*P* < 0.05). The results show that the cAMP concentration was significantly lower in the LSW-treated samples than in the control but higher in the HSW-treated group.

With the results of HdNKA enzyme activity and expression level (q-PCR) and the cAMP concentration in mantle and gill, we investigated the correlation between cAMP concentration, HdNKA activity and *Hdnka* α and β subunit mRNA expression levels. Statistical analysis showed that the cAMP concentration was moderately correlated with the *Hdnka* mRNA expression level (of both the α and β subunits) and the HdNKA activity, including in samples of mantle and gill (*P* < 0.0005) (**Figures 9C–F**).

## Discussion

One of the main objectives of the present study was to determine the role of the osmotic pressure regulation in Pacific abalone. The second objective was to investigate the possible mechanisms of HdNKA involved in this process. Therefore, the expression and activities of HdNKA in tissue were measured after treatment with either LSW or HSW.

Pacific abalone belongs to the mollusk phylum. Similar to other invertebrates, it has an open circulatory system. In this system, fluid in a cavity called the hemocoel bathes the organs directly with oxygen and nutrients, and there is no distinction between blood and interstitial fluid (Monahan-Earley et al., 2013). This combined fluid is called hemolymph. Hemolymph is composed of water, inorganic salts (mostly sodium, chlorine, potassium and calcium), and organic compounds (mostly carbohydrates, proteins, and lipids) (Wyatt, 1961; Richards and Davies, 1977; Sowers et al., 2006). Hemolymph is analogous to the blood in vertebrates, which circulates in the interior of the body in direct contact with the animal’s tissues. This arrangement means that all cells are surrounded by hemolymph. The ions in hemolymph act directly on the surfaces of the tissue cells. From the ion concentrations in hemolymph, we could find that salinity significant influenced the concentrations of Na^+^ and K^+^ in a clear time-dependent manner after sudden treatment with LSW. Moreover, with the progression of the treatment, the ion concentration remained at a low level. In contrast, the concentrations of these ions greatly increased when treated with HSW. These data were consistent with previous research in *Haliotis diversicolor supertexta* (Cheng et al., 2002). Sudden salinity changes directly changed the ion concentrations that surrounded tissue, and the balance between intracellular and extracellular electrical potentials was altered at the initial time. This sudden change may activate the stress response of the organism. Ion concentrations may initially decrease after LSW treatment and increase after HSW treatment. When compared the ion concentrations in tissues, both the Na^+^ and K^+^ were basically maintained at the same level respectively (**Figures 2D,E**). Due to the complexity of cell osmoregulation, this stasis may be affected by the change in cell volume and the transmembrane transport of water molecules. Moreover, both the Na^+^ and K^+^ could be transported separately by several ion channels. There are also several channel proteins that can cotransport both Na^+^ and K^+^ at the same time. As an important ion transporter, the function of NKA in maintaining the ion concentration at a well-balanced level in Pacific abalone has never been reported.

Thus, our initial attempts were directed toward cloning the full-length cDNAs of the *Hdnka* α and β subunits. In the present study, only one α subunit and one β subunit of *Hdnka* cDNAs were identified from the Pacific abalone. Multiple sequence alignments showed that the amino acid sequence of the *Hdnka* α subunit had high similarity with those of other species. This result demonstrates that the gene is much conserved in evolution. The subunit composition of the NKA is tissue specific (Therien and Blostein, 1999). Nevertheless, its α subunit, the catalytic subunit, is common to all of the subtypes and possesses high sequence similarity with α subunits of other P-type ATPases, even the sarco/endoplasmic reticulum Ca^2+^-ATPase (SERCA) and the gastric H^+^/K^+^-ATPase (Møller et al., 1996). This enzyme catalyzes the same reaction in different species. The conserved domain of the α subunit is the active site region of the enzyme. However, multiple sequence alignments of the β subunit amino acid sequences show that the sequences of this gene and those of other species share low similarity. The main function of NKA is pumping sodium out of cells while pumping potassium into cells via binding sites on the α subunit. Highly conserved amino acids sequences are thought to have functional value. Results of bioinformatics analysis indicated that the α subunit is the catalytic subunit of the NKA on another perspective.

Subunit α of NKA is the catalytic subunit. Therefore, most previous studies have particularly focused on this subunit in other species. The regulation of NKA under osmotic stress has been determined using the renal epithelial cell line NBL-1 (Ferrer-Martínez et al., 1996). This study indicated that the hypertonic shock increased the amount of NKA α subunit mRNA but had no influence on the β subunit levels. In addition, this stimulation was not correlated with significant changes in the amount of NKA α subunit protein. From the q-PCR data in our experiment, we found that after the treatment with LSW, the change in *Hdnka* α subunit expression could be divided into three stages. In the first phase, the expression slightly increased in both mantle (*P >* 0.05) and gill (*P* < 0.05). This difference indicates that gill is more sensitive when faced with salinity change. Moreover, the first phase is very ephemeral. This transient increase may due to the stress response of organism, which resulted from the sudden decrease of ion concentration in hemolymph. The rapid change resulted in electrical potential and altered the expression of NKA at the initial time. In the second phase, there is a rapid reduction of α subunit expression in these two tissues. To keep the normal biological function of the organism, the intracellular ion concentration needs to be maintained within a certain physiological range, which leads to the significant reduction in NKA expression in later stage. In the last phase, expression increases slightly. In contrast, the HSW treatment led to a slight reduction in expression at the first stage. However, this period lasted longer than it did during the LSW treatment. These data indicated that Pacific abalone was more sensitive to low salinity than to high salinity. Then, there is a significant increase in expression. NKA α subunit expression changes in the euryhaline teleost *Fundulus heteroclitus* was also been divided into two phases in a previous publication: a stress period and an adjustment period. There were significant differences in the activity of NKA between these two periods (Mancera and McCormick, 2000). Moreover, there are many previous studies that showed that a change in salinity has no influence on the activity of NKA (Heijden et al., 1997; Kelly et al., 1999; Romao et al., 2001). These differences may due to the differences in the adaptability of different species to osmotic changes and their different modes of regulating osmotic pressure.

There is much less studies on the β subunit of NKA than on the NKA α subunit due to its lack of catalytic activity. Most of the research is on fish. Moreover, the conclusions about the effect of salinity on the β subunit are also inconsistent (Blasiole et al., 2003; Deane and Woo, 2005; Fiol et al., 2006). From the data of our experiment, we found that the influence of salinity changes on the expression of the *Hdnka* β subunit was consistent with the changes in the expression of the *Hdnka* α subunit. Furthermore, we also determined the expression of HdNKA at the protein level by western blot. Like the results from q-PCR, the HSW treatment increased the expression of HdNKA, and LSW treatment led a reduction in HdNKA expression. In contrast, the amount of NKA α subunit protein was not modified after hypertonic shock at any time tested, even when the biological activity of the pump was already enhanced, as in a previous study (Ferrer-Martínez et al., 1996). In addition, the activity of HdNKA was measured in our experiment. The activity of NKA in mantle was increased significantly by 3 h after transfer into HSW, but the levels of both mRNA (both α and β) and protein were not changed at the same time point (**Figures 8A,G**). The response of NKA activity to the salinity changes occurred a little earlier than that of gene expression. These data indicated that the increase in NKA activity is not due to only the accumulation of NKA. There may be some other factors. In gill, the protein abundance of the α subunit was slightly enhanced at 3 h after transfer into HSW (**Figure 8H**), but the patterns were different from those of the mRNA expression levels of α and β (non-changed; **Figures 8B,D**). These differences may be caused by the accuracy of the detection method. From these data, we concluded that 3–6 h is the critical time range. Due to the subtle effects of individual differences, the mix in protein samples may cause the slight increase at the 3 h time point. In summary, these data indicated that HdNKA was significantly regulated by the salinity change at both the mRNA and protein levels in Pacific abalone. The regulation process in Pacific abalone when faced with salinity changes mainly includes three stages: a passive stress response period, an active adjustment period and an adaptation period.

The regulation of NKA was divided into two categories, endogenous and exogenous. As an intracellular second messenger, cAMP could regulate the activity of NKA (Kiroytcheva et al., 1999). In conclusion, substances causing an increase in cAMP upregulate the activity of NKA. This study indicated that cAMP could regulate the NKA by PKA phosphorylation on the NKA α subunit in rat. However, in another study, the activity of NKA in rat was inhibited by cAMP (Kurihara et al., 2000). This research suggested that NKA activity could be regulated by a cAMP-dependent protein kinase that is anchored on the membrane via its anchoring protein. Unfortunately, both of these two reports did not determine the influence of cAMP on the expression of NKA at the mRNA level. Currently, little is known about whether salinity changes affect the concentration of cAMP in Pacific abalone. Moreover, the effect of cAMP on the expression of NKA is still unknown. Sommer and Mantel (1991)have reported that short-term acclimation of crabs to 40% seawater increased the cAMP content of the posterior gills from its value in the controls. Nonetheless, this result was not consistent with our findings. In the present study, the concentration of cAMP was measured after sudden treatment with LSW or HSW at different times. In both mantle and gill, the cAMP concentration was substantially lower after sudden treatment with LSW. In contrast, the HSW treatment increased the cAMP concentration rapidly. These findings suggested that a cAMP generation system is stimulated after salinity changes. The differences in the influence of salinity changes on the cAMP concentration may due to the differences between species. In addition, statistical analysis showed that the cAMP concentration was highly significant and positively correlated with the HdNKA activity and HdNKA expression in both mantle and gill samples in Pacific abalone.

In summary, the present study has identified the full-length of *Hdnka* α and β subunits in Pacific abalone *H. discus hannai*. From the data of our experiment, we found that NKA may play an important role during the process of osmotic pressure regulation in Pacific abalone. When faced with sudden salinity change, the responses of *Hdnka* α subunit could be divided into three stages: stress period, adjustment period and stable period. Moreover, the concentration of cAMP in mantle and gill were affected by salinity change. Changes in salinity may affect the synthesis of cAMP and then affect the transcription, translation, and enzyme activity of HdNKA. Further research is needed to determine whether salinity changes directly affect the HdNKA activity by the phosphorylation of PKA through the cAMP pathway in Pacific abalone. In addition, the role of the significant changes in non-catalytic subunit (the *Hdnka* β subunit) expression needs further investigation.

## Author Contributions

XL conceived and supervised the study. YJ performed most of the experiments and prepared the figures. YJ and XL analyzed the data and wrote the paper.

## Conflict of Interest Statement

The authors declare that the research was conducted in the absence of any commercial or financial relationships that could be construed as a potential conflict of interest.

## Footnotes

1 http://www.cbs.dtu.dk/services/TMHMM
