# Supplementary material for: Expression of Na+/K+-ATPase Was Affected by Salinity Change in Pacific abalone Haliotis discus hannai
Source: Front Physiol. 2018 Sep 7;9:1244. doi: 10.3389/fphys.2018.01244 (PMC6137147; doi:10.3389/fphys.2018.01244)
Supplement: Supplementary file 1 [file Data_Sheet_1.DOC]

**Figure legends**

**Figure S1:** Specificity and efficiency of the primers and antibodies. (A) Melt curve plot of NKA α subunit. (B) Melt curve plot of NKA β subunit. (C) Melt curve plot of actb. (D) Efficiency of the primers used for q-PCR. (E) Influence of salinity change on the expression of actb. (F) Expression of actb in different tissues. (G) Antigen information of the primary antibodies (H) Specificity of the antibodies.

**The accession numbers and amino acid sequence of all used sequences**

**Na^+^/K^+^-ATPase α subunit**

>Haliotis Discus Hannai MG767304

MASSKVSKSSQPRAESYRYAVTPSDNDDKKKSKKEKKKENLDELKQELEMEEHKIPLEELYERLGSDPNMGHSVEKAKEILLRDGPNMLTPPKTTPEWVKFCKTLFGGFSLLLWIGAILCYIAYSIQAGAMEDPPGDNLYLGIVLTAVVVVTGCFSYYQEAKSSKIMDSFKNMVPQYAVAIRGGQKHNIHAEELVLGDIIDVKFGDRVPADIRVITAHSFKVDNSSLTGESEPQTRTADFTHENPLETRNLAFFSTNAVEGDCRGIVVRTGDKTVMGRIANLASGLEVGETPIAKEIAHFIHIITGVAVFLGVSFFVIAFILGYFWLDAVIFLIGIIVANVPEGLLATVTVCLTLTAKRMASKNCLVKNLEAVETLGSTSTICSDKTGTLTQNRMTVAHMWFDGRIVEADTSDDQSNATYSRTDLTWMNLARVAMLCNRAEFKLGQDDTPILKRECNGDASESALLKCVELSIGNVTEFRRRNKKVTEIPFNSTNKYQVSIHETEDPNDPRYLLVMKGAPERIMDRCSTVILNGKEQPIDDSFREAFNAAYLELGGLGERVLGFCDYFLPIDQFAPGFSFDPDGPNFPITGLRFVGLMAMIDPPRAAVPDAVGKCRSAGIKVIMVTGDHPITAKAIAKGVGIISEGSKTVEDIAAERGIPVEEVDPREAKAAVVHGADLRDMTPAQIDEILRNHGEIVFARTSPQQKLIIVEGCQRQGAIVAVTGDGVNDSPALKKADIGVAMGIAGSDVSKQAADMILLDDNFASIVTGVEEGRLIFDNLKKSIAYTLTSNIPEISPFLLFILADIPLPLGTITILCIDLGTDMVPAISLAYEQAESDIMKRQPRDPVKDKLVNERLISMAYGQIGMIQASAGFFVYFVIMGENGFWMSKLLGIREEWDSLGINDLEDSYGQEWTYSQRKRLEYTCHTAFFVSIVIVQWADLIICKTRRLSLFQQGMKNHRLSFGIFFETALAAFLTYCPGLDQGLRMQHLRLSWWFPAMPFSLAIFIYDECRKFILRRNPGGFVERETYY

>Biomphalaria glabrata XP_013071165.1

MASSKVNQKGGPRAESYRYAVTPSGDDKKKKKKKGEEKLDELKQELDMDEHKIPIEELYERTGADPNNGHSVERAKEILARDGPNQLTPPKTTPEWVKFCKVLFTGFSLLLWIGAILCYIAYSIQASQAEDPPGDNLYLGIVLTAVVVVTGCFSYYQEAKSSRIMDSFKNMVPQYAIVVRGGQKLSIRAEELVLGDIVEVKFGDRVPADIRVISAHGFKVDNSSLTGESEPQTRTAEFTHENPLETRNLAFFSTNAVEGTCRGIVVKTGDNSVMGRIANLASGLEVGETPIAKEIAHFIHIITGVAVFLGVTFFIIAFILGYFWLDAVIFLIGIIVANVPEGLLATVTVCLTLTAKRMAKKNCLVKNLEAVETLGSTSTICSDKTGTLTQNRMTVAHMWFDGRIIEADTSDDQSNASYSRNDPTWMSLARIGMLCNRAEFKVGQENVPVLKRECNGDASESALLKCVELSIGNVTEFRRRNKKISEIPFNSTNKYQVSIHETEDPNDPSFLLVMKGAPERIMDRCSTILMHGKTQPLDDNFRDAFNVAYLELGGLGERVLGFCDYVLPSSEFPPNYQFDPEGPNFPITGLRFVGLMSMIDPPRAAVPDAVGKCRSAGIKVIMVTGDHPITAKAIAKGVGIISEGSKTVEDIAAERGCPVEEVDPREAKAAVIHGGDLRDMTPAQIDEILINHSEIVFARTSPQQKLIIVEGCQRQGQIVAVTGDGVNDSPALKKADIGVAMGIAGSDVSKQAADMILLDDNFASIVTGVEEGRLIFDNLKKSIAYTLTSNIPEISPFLLFILADIPLPLGTITILCIDLGTDMVPAISLAYEQAELDIMKRLPRNPLKDKLVNDRLISMAYGQIGMIQATAGFFTYFVIMGENGFWMSRLLGIRKEWDSLGINDLEDSYGQEWTYSQRKKLEYTCHTAFFVSIVIVQWADLIICKTRRLSLFQQGMKNHRLTFGLFFETALAAFLCYCPGLDKGLRMQPLRFTWWLVALPYSLAIFIYDEVRKFILRRHPGGFVERETYY

>Lottia gigantean XP_009065938.1

MPRAESYRYAVTPSTDDKKKKKKKGKEDLDELKQELTMDEHKLPIEELYERLTTDPNMGLTVERAREILLRDGPNCLTPPKTTPEWVKFCKQLFGGFSLLLWIGAILCFIAYSIQASALEYPPGDNLYLGIVLAAVVIVTGCFSYYQEAKSSRIMDSFKNMVPQYAVCIRSGDKLNLKAEELVVGDVIDIKFGDRVPGDIRVISAHGFKVDNSSLTGESEPQTRTAEFTHENPLETRNLAFFSTNAVEGTCRGVVVKTGDKTVMGRIANLASGLEVGETPIAKEIAHFIHLITGVAVFLGVTFFIIAFILGYYWLDAVIFLIGIIVANVPEGLLATVTVCLTLTAKRMARKNCLVKNLEAVETLGSTSTICSDKTGTLTQNRMTVAHMWFDGHILEADTTDDQSNAAYSRNDPTWNALARIAMLCNRAEFKTGQESVPILKKECNGDASESALLKCVELSIGNVTEYRRRNKKICEIPFNSTNKYQLSIHETEDPNDPRMILVMKGAPERIMDRCSTVLLNGKETPLDDNFREAFNAAYLELGGLGERVLGFCDYVLPNEQYPPGYEFDPEGPNFPITGLRFVGLMSMIDPPRAAVPDAVGKCRSAGIKVIMVTGDHPITAKAIAKGVGIISEGSKTVEDVASERGIPVEEVDPRDAKAAVIHGQDLRDMTPAQIDEILRNHNEIVFARTSPQQKLIIVEGCQRQGAIVAVTGDGVNDSPALKKADIGVAMGISGSDVSKQAADMILLDDNFASIVTGVEEGRLIFDNLKKSIAYTLTSNIPEISPFLFFILLDIPLPLGTITILCIDLGTDMVPAISLAYEQAENDIMKRQPRDPLNDKLVNERLISMAYGQIGMIQASAGFFVYFVIHGENGFWISRLMGIREAWDSRGINDLEDSYGQEWTYHQRKVLEYTTHTAFFVSIVIVQWADLIICKTRRLSLFQQGMKNHRLTFGIFFETALAIFLTYCPGLDQGLRMQHLRFTWWLPALPFSLAIFVYDECRKYILRRRPGGFVERETYY

>Aplysia californica XP_005093050.1

MASSKVNKQGPRADSYRYAVTPSGEEKKKKKKKKGDDDLDALKQELEMDEHKVPIEELYDRLEADPTNGHSPDRAKEILARDGPNMLTPPKTTPEWIKFCKVLFTGFSLLLWIGAILCYIAYSIQASQQENPPGDNLYLGIVLTAVVVVTGCFSYYQEAKSSRIMDSFKNMVPQYAIVVRGSQKLSVRAEELVLGDIIEVKFGDRVPADMRVISAHGFKVDNSSLTGESEPQSRTAEFTHENPLETRNLAFFSTNAVEGTARGIVVKIGDQSVMGRIANLASGLEVNETPIAKEIAHFIHIITGVAVFLGVTFFIIAFILGYFWLDAVIFLIGIIVANVPEGLLATVTVCLTLTAKRMAKKNCLVKNLEAVETLGSTSTICSDKTGTLTQNRMTVAHMWFDGRISEAETNEDQTTASYSSKDPTWMSLARIGMLCNRAEFKANQENVPVLKRECNGDASESALLKCVELNIGKVTEFRRRNKKIVEIPFNSTNKYQVSIHETEDPNDPSYLLVMKGAPERIMDRCSTILMHGKDQPLDDNFREAFNAAYLELGGLGERVLGFCDYNLPIDEFPPNFEFDAEGPNFPITGLRFVGLMSMIDPPRAAVPDAVGKCRSAGIKVIMVTGDHPITAKAIAKGVGIISEGSKTVEDLAAERSVPVEEVDPREAKAAVIHGGDLRDMTPAQIDEILINHAEIVFARTSPQQKLIIVEGCQRQGQIVAVTGDGVNDSPALKKADIGVAMGIAGSDVSKQAADMILLDDNFASIVTGVEEGRLIFDNLKKSIAYTLTSNIPEISPFLLFILADIPLPLGTITILCIDLGTDMVPAISLAYEQAELDIMKRAPRNPFTDKLVNERLISMAYGQIGMIQASAGFFTYFVIMGENGFWMSTLLGIRKEWDSLGINDLEDSYGQEWTYSQRKKLEYTCHTAFFVAIVVVQWADLIICKTRRLSLFQQGMKNHRLTFGLFFETALAAFLCYCPGLDKGLRMQPLRFTWWLTAIPYSLTIFIYDECRKLILRRNPGGFVERETYY

>Doryteuthis opalescens ABO61333.1

MASSKAKSQQAPRADSYRYAVTPGGQEKKKKKKGEDLDELKQELDMDEHKIPIEELYRRYGTDPTTGLSPEKAKEILLRDGPNCLTPPKTTPEWVKFCKTLFGGFSMLLWIGAILCFIAYGIQAGTYDDPPGDNLYLGIVLTAVVIVTGIFSYYQEAKSSKIMDSFKNMVPQYAVVVRSGEKLNVRAEELVVGDVVEVKFGDRIPSDIRVISAHSFKVDNSSLTGESEPQSRSAEFTNENPLETKNLAFFSTNAVEGTCVGLVVKTGDKTVMGRIANLASGLEVGETPIAKEIAHFIHLITGVAVFLGVTFFVIAFILGYYWLDAVIFLIGIIVANVPEGLLATVTVCLTLTAKRMARKNCLVKNLEAVETLGSTSTICSDKTGTLTQNRMTVAHMWYGGSIVEADTSEDQSNATYNKDTADWIALSRIAMLCNRAEFKAGQDNVPVLKKECNGDASESALLKCVELSIGNVPEYRRRNKKVVEIPFNSTNKYQVSIHENEDPNDPRYILVMKGAPERIMDRCSTALMNGKDLDVDEPFRTNFNAAYMELGGLGERVLGFCDYILPTESFPPGFQFDGDEVNFPLTGLRFVGLMSMIDPPRAAVPDAVGKCRSAGIKVIMVTGDHPITAKAIAKGVGIISEGSKTVEDISAEQGIPVDQVNSGDAGAAVIHGTDLRDMTPAQIDEILRNHSEIVFARTSPQQKLIIVEGCQRQGHIVAVTGDGVNDSPALKKADIGVAMGIAGSDVSKQAADMILLDDNFASIVTGVEEGRLIFDNLKKSIAYTLTSNIPEISPFLFFILLDIPLPLGTITILCIDLGTDMVPAISLAYEQAESDIMKRQPRDPVNDKLVNERLISMAYGQIGMIQASAGFFTYFVIMAENGFWISHLLGIRKNWDSMGVNDLEDSYGQEWTYAQRKKLEYTCHTAFFVSIVVVQWADLIICKTRRLSLFQQGMKNHRLTFGLIFETVLACFLTYCPGLDQGLRMQPLRASWWFPAFPYSLLIFIYDECRKLILRRHPGGWVENETYY

>Octopus bimaculatus AEH68837.1

MASSKVKSQQAPRAESYRYAVTPGGQEKKKGKKKGEDLDELKQELDMDEHKVSIEELYQRLGTDPTRGLSPERAKEILLRDGPNCLTPPKTTPEWVKFCKTLFGGFSMLLWIGAILCFIAYGIQAGTYDDPPGDNLYLGIVLTAVVVVTGIFSYYQEAKSSRIMDSFKNMVPQYAVVLRNGEKLNVHAEDLVVGDVVDVKFGDRVPADIRVISAHSFKVDNSSLTGESEPQSRSAEFTNENPLETKNLAFFSTNAVEGTCLGLVIKTGDKTVMGRIANLASGLEVGETPIAKEIAHFIHLITGVAVFLGVTFFVIAFILGYFWLDAVIFLIGIIVANVPEGLLATVTVCLTLTAKRMARKNCLVKNLEAVETLGSTSTICSDKTGTLTQNRMTVAHMWYGGKIVEADTSEDQSNATYNKDNVDWKALSRIAMLCNRAEFKAGQDGVPVLKRECNGDASESALLKCVELSIGGVPEYRRRNKKVVEIPFNSTNKYQVSIHNNEDPNDPCYFLVMKGAPERIMERCTVALVNGKEMTIDESFKNDFNTAYMELGGLGERVLGFCDYTLPTESFPPGFQFDGDEVNFPLTGLRFVGLMSMIDPPRAAVPDAVGKCRSAGIKVIMVTGDHPITAKAIAKGVGIISEGSKTVEDLAAEQGVAVDQVNPRDAKAAVIHGSDLRDMTPAQIDEILRNHSEIVFARTSPQQKLIIVEGCQRQGQIVAVTGDGVNDSPALKKADIGVAMGIAGSDVSKQAADMILLDDNFASIVTGVEEGRLIFDNLKKSIAYTLTSNIPEISPFLFFILLDIPLPLGTITILCIDLGTDMVPAISLAYEQAESDIMKRQPRDPVNDKLVNERLISMAYGQIGMIQASAGFFTYFVIMAENGFWMSRLLGLRKNWDSMGVNDLEDSYGQEWTYSQRKKLEYTCHTAFFVSIVVVQWADLIICKTRRLSLFQQGMKNHRLTFGLFFETVLAAFLTYCPGLDQGLRMQPLRLSWWFPAFPYSLTIFIYDECRKFILRRNPGGWVEYETYY

>Crassostrea gigas XP_011441273.2

MSPMSVVVSSGKVVFKRAILYKQREFLSVTAWAEMASTKVKSQQQNPRADSYRYAVTPGGEDPKKGKKKKSKKENLDELKQELEMDEHKVPIEELYARLGSDPSMGLTSQRAKEILERDGPNALTPPPTTPEWVKFCKLLFGGFSLLLWIGAILCFIAYSIQASAYDDPPGDNLYLGIVLTAVVLVTGIFSYYQEAKSSKIMESFKSMVPQFAVVTRNGKISNIKAEELVVGDVIDVKFGDRVPADVRVITAHGFKVDNSSLTGESEPQTRTADFTNDNPLETRNIAFFSTNAVEGTCRGIVIRCGDNTVMGRIANLASGLEVGETPIAKEIAHFIHIVTGVAVFLGVTFFIIAFILEYFWLDAVIFLIGIIVANVPEGLLATVTVCLTLTAKRMAKKNCLVKNLEAVETLGSTSTICSDKTGTLTQNRMTVAHMWFDGKIVEADTSDDQTNAAYSGSDETWMALARVAMLCNRAEFTANQEHLPVLKRECNGDASESALLKCVELSIGKVTEFRNKNKKICEIPFNSTNKYQVSIHETENPNDARCILVMKGAPERILERCSTILMHGKEVPMDDNFREAFNNAYMELGGLGERVLGFCDYFLPSDQYPPGYPYDSDDANFPLTGLRFVGLMSMIDPPRAAVPDAVGKCRSAGIKVIMVTGDHPITAKAIAKGVGIISEGSKTIEDIAAERGVPVEEIQDTSAAKAAVIHGSDLRDMTPAQIDEVLKNHSEIVFARTSPQQKLIIVEGCQRQGAIVAVTGDGVNDSPALKKADIGVAMGIAGSDVSKQAADMILLDDNFASIVTGVEEGRLIFDNLKKSIAYTLTSNIPEISPFLFFILLDIPLPLGTITILCIDLGTDMVPAISMAYEGAESDIMKRQPRDPFKDKLVNERLISMAYGQIGMIQASSGFFVYFVIMGENGFWMTRLLGIREQWDSQAVNDLQDSYGQEWTYNQRKILEYTCHTAFFVSIVVVQWADLIICKTRRLSLFHQGMKNHHMTFGLFFETALAAFLTYCPGLEQGLRMQNLRWSWWFPAFPFSIAIFIYDESRKYILRRNPGGFVERETYY

>Lingula anatine XP_013379613.1

MASKKLQAQQQDDTERSDSYRFAVKQADAEKDPTALSTSGKSGKKKGKKGKKQDMEELKQELEMDEHRISIEELYDRLGADPNNGHTSERAKEYLERDGPNELSPPKTTPEWVKFCKQLFGGFALLLWIGAILCFIAYSIQSTQYDIPPGDNLYLGIVLTAVVVVTGCFSYYQEAKSSRIMDSFKEMIPQYALVKRDGQLIEVRAETLVVGDIIEVKFGDRVPADIRVIKAHGFKVDNSSLTGESEPQTRLPEFTSENPLETRNLAFFSTNAVEGTCTGIVVSIGDRTVMGRIANLASGLEMGETPIAREIEHFIHIITGVAVFLGVSFFIIAFILGYYWLDAVIFLIGIIVANVPEGLLATVTVCLTLTAKRMANKNCLVKNLEAVETLGSTSTICSDKTGTLTQNRMTVAHMWFDDRIVEADTSDDQSSATYNKDTPTWTSLSRVAMLCNRASFKVGEEEKPVLKRECNGDASESALLKCVELSVGGVTEFREKNPKVCEIPFNSSNKYQVSIHETSDPDDPRYILVMKGAPERILDRCTTIMINGEEVPLDDQKKEDFNQAYLELGGLGERVLGFCDYFLPADKFPPGYAFDSDDPNFPLTGLRFIGLMSMIDPPRAAVPDAVGKCRSAGIKVIMVTGDHPITAKAIAKGVGIISEGNETVEDIAAREGIPVSEVDPSRAKACVVHGMDLRDMTPFQIDEILRNHTEIVFARTSPQQKLIIVEGCQRQGAIVAVTGDGVNDSPALKKADIGVAMGIAGSDVSKQAADMVLLDDNFASIVTGVEEGRLIFDNLKKSIAYTLTSNIPEITPFLFFICLDIPLPLGTITILCIDLGTDMVPAISLAYEKAESDIMKRQPRDPKHDKLVNQRLISMAYGQIGMMQASAGFFTYFVIMGENGFWPNHLIGIRKEWDSKSINDLEDSYGQEWTYAQRKMLEYTCHTAFFITIVIVQWADLIICKTRRNSIVEQGMSNHYMTFALFFETCLAAFLSYCPGLDKGLRMFPLRFMWWLPAFPFSAAIWIYDEARRFFMRRSPGGFIEQETYY

>Capitella teleta ELU12040.1

MSSKEEPRSDSYRYAVQPKSEEAAGAQQKKKKTKKNKKAELDDLKQELDMDDHKIPLEELYARHSVDPIKGHTTECARAFLERDGPNELSPPKTTPEWVKFCKQLFGGFSMLLWLGAILCFIAYSIQASTYEDPAGDNLYLGIVLTAVVVVTGIFAYYQEAKSSKIMESFKNLVPQFALVIRNGEKLNLHAEELVVGDIIEVKFGDRVPADVRVISAHGFKVDNSSLTGESEPQSRSSDFTHENPLETRNLAFFSTNAVEGTMRGIVVSTGDRTVMGRIANLASGLEVGETPIAREIGHFIHIITGVAVFLGVTFFIIAFILGYYWLDAVIFLIGIIVANVPEGLLATVTVCLTLTAKRMASKNCLVKNLEAVETLGSTSTICSDKTGTLTQNRMTVAHMWFDNRIVEADTSEDQTSATYDGESVTWMALARICMLCNRAEFKSGQENIPVLKRECNGDASESALLKCVELSIGHVCEFREKNRKVIEIPFNSSNKYQVSVHETDIEGDDRYILVMKGAPERILDRCSTILINGEERALDEEWRDAFNQSYLELGGLGERVLGFCDYFLPLDQFPVGYPFDADEENFPLTGLRFVGLLSMIDPPRAAVPDAVGKCRSAGIKVIMVTGDHPITAKAIAKGVGIISEGNETVEDIAARLGVPVSEVNPRDAHAVVVHGSDLRDMTPAQIDDILANHSEIVFARTSPQQKLIIVEGCQRQGQIVAVTGDGVNDSPALKKADIGVAMGIAGSDVSKQAADMILLDDNFASIVTGVEEGRLIFDNLKKSIAYTLTSNIPEISPFLIFILADVPLPLGTITILCIDLGTDLVPAISLAYERAESDIMKRMPRDPLNDKLVNSRLIGMAYGQIGMIQASAGFFVYFVIMAENGFWPSRLLGLRKAWDSKGVNDLEDSHGQEWTYAQRKVLEYTCHTAFFMSIVIVQWADLMICKTRRNSIIHQGMTNHHLTFGLFFETALAAFMAYCPGLDKGLRMYPLRWTWWIVPMPFSLVIFIYDEGRKYLLRKNPGGWVETETYY

>Homo sapiens NP_000692.2

MGKGVGRDKYEPAAVSEQGDKKGKKGKKDRDMDELKKEVSMDDHKLSLDELHRKYGTDLSRGLTSARAAEILARDGPNALTPPPTTPEWIKFCRQLFGGFSMLLWIGAILCFLAYSIQAATEEEPQNDNLYLGVVLSAVVIITGCFSYYQEAKSSKIMESFKNMVPQQALVIRNGEKMSINAEEVVVGDLVEVKGGDRIPADLRIISANGCKVDNSSLTGESEPQTRSPDFTNENPLETRNIAFFSTNCVEGTARGIVVYTGDRTVMGRIATLASGLEGGQTPIAAEIEHFIHIITGVAVFLGVSFFILSLILEYTWLEAVIFLIGIIVANVPEGLLATVTVCLTLTAKRMARKNCLVKNLEAVETLGSTSTICSDKTGTLTQNRMTVAHMWFDNQIHEADTTENQSGVSFDKTSATWLALSRIAGLCNRAVFQANQENLPILKRAVAGDASESALLKCIELCCGSVKEMRERYAKIVEIPFNSTNKYQLSIHKNPNTSEPQHLLVMKGAPERILDRCSSILLHGKEQPLDEELKDAFQNAYLELGGLGERVLGFCHLFLPDEQFPEGFQFDTDDVNFPIDNLCFVGLISMIDPPRAAVPDAVGKCRSAGIKVIMVTGDHPITAKAIAKGVGIISEGNETVEDIAARLNIPVSQVNPRDAKACVVHGSDLKDMTSEQLDDILKYHTEIVFARTSPQQKLIIVEGCQRQGAIVAVTGDGVNDSPALKKADIGVAMGIAGSDVSKQAADMILLDDNFASIVTGVEEGRLIFDNLKKSIAYTLTSNIPEITPFLIFIIANIPLPLGTVTILCIDLGTDMVPAISLAYEQAESDIMKRQPRNPKTDKLVNERLISMAYGQIGMIQALGGFFTYFVILAENGFLPIHLLGLRVDWDDRWINDVEDSYGQQWTYEQRKIVEFTCHTAFFVSIVVVQWADLVICKTRRNSVFQQGMKNKILIFGLFEETALAAFLSYCPGMGVALRMYPLKPTWWFCAFPYSLLIFVYDEVRKLIIRRRPGGWVEKETYY

>Portunus trituberculatus AGF90965.1

MADTGRTDSYRHATDRNVPDDNRTVKGDPKSKKKNVKGKRKGEKEKDMDNLKQELELDEHKVPIEELFQRLSVNPDTGLTQAEARRRLERDGPNALTPPKQTPEWVKFCKNLFGGFSLLLWIGAILCFIAYSIEAASEEEPNNDNLYLGIVLTAVVIITGIFSYYQESKSSRIMESFKNLVPQYAIVIREGEKLNVQAEELCIGDIIDVKFGDRIPADMRVIEARGFKVDNSSLTGESEPQSRSPEFTSENPLETKNLAFFSTNAVEGTCKGIVINIGDNTVMGRIAGLASGLETGETPIAKEISHFIHIITGVAVFLGVTFFVIAFILGYHWLDAVVFLIGIIVANVPEGLLATVTVCLTLTAKRMAAKNCLVKNLEAVETLGSTSTICSDKTGTLTQNRMTVAHMWFDNTIIEADTSEDQSGCQYDKTSEGWKALSRIAALCNRAEFKTGQEDVPILKREVNGDASEAALLKCVELAIGDVRGWRSRNKKVCEIPFNSTNKYQVSIHETQDKNDLRYLLVMKGAPERILERCSTIFINGEEKSLDEEMKESFNNAYLELGGLGERVLGFCDYVLPSDKYPLGYPFDADAVNFPVHGLRFVGLMSMIDPPRAAVPDAVAKCRSAGIKVIMVTGDHPITAKAIAKSVGIISEGNETVEDIAQRLNIPIKEVDPREAKAAVVHGSELRDMTSEQLDDVLIHHTEIVFARTSPQQKLIIVEGCQRMGAIVAVTGDGVNDSPALKKADIGVAMGIAGSDVSKQAADMILLDDNFASIVTGVEEGRLIFDNLKKSIAYTLTSNIPEISPFLFFMIASVPLPLGTVTILCIDLGTDMVPAISLAYEEAESDIMKRQPRNPFTDKLVNERLISMAYGQIGMIQALAGFYVYFVIMAENGFLPPILFGIREQWDSKAINDLEDYYGQEWTYHDRKILEYTCHTAFFVAIVVVQWADLIICKTRRNSILHQGMKNMVLNFGLCFETTLAAFLSYTPGMDKGLRMYPLKFYWWLPALPFSLLIFVYDECRRFVLRRNPGGWVEMETYY

>Penaeus monodon AGV55413.1

MADSKKKPQKAKGKKGDKDLNDLKQELELDEHKVPIEELFQRLTVNPDTGLSQSEAKRRIERDGPNALTPPKQTPEWVKFCKNLFGGFSLLLWIGAILCFIAYSIETAAEEEPNKDNLYLGIVLTAVVIITGVFSYYQESKSSRIMESFKNMVPQYAIVLRDGEKQNVQAEELCIGDIVEVKFGDRIPADIRVIESRGFKVDNSSLTGESEPQSRSSEYTSENPLETKNLAFFSTNAVEGTCKGVVIMIGDNTVMGRIAGLASGLKTGETPIAKEITHFIHIITGVAVFLGVTFFVIAFILGYHWLDAVVFLIGIIVANVPEGLLATVTVCLTLTAKRMAAKNCLVKNLEAVETLGSTSTICSDKTGTLTQNRMTVAHMWFDNTIIEADTSEDQSGCQYDKTSQGWKALSRIAALCNRAEFKSGMENTPILKREVNGDASEAALLKCVELAVGDVKGWRARNKKVCEIPFNSTNKYQVSIHNTEDKNDPRYLLVMKGAPERILERCSTIYINGEEKALDEEMKEAFNNAYLELGGLGERVLGLCDYVLPTDKYPHGYPFDADAVNFPVHGLRFVGLMSMIDPPRAAVPDAVAKCRSAGIKVIMVTGDHPITAKAIAKSVGIISEGNETVEDIAQRLNIPIKEVDPTEAKAAVVHGSELRDMTSEQLDDVLLHHTEIVFARTSPQQKLIIVEGCQRMGAIVAVTGDGVNDSPALKKADIGVAMGIAGSDVSKQAADMILLDDNFASIVTGVEEGRLIFDNLKKSIAYTLTSNIPEISPFLFFMIASVPLPLGTVTILCIDLGTDMVPAISLAYEEAKSDIMKRQPRNPFTDKLVNERLISMAYGQIGMIQALAGFFTYFVIMAENGFLPPHLFGLRERWDSKAINDLEDHYGQEWTFHDRKILEYTCHTAFFTSIVIVQWADLIICKTRRNSIVHQGMKNWVLNFGLVFETTLAAFLSYTPGMDKGLRMYPLKFYWWLPALPFSLLIFIYDEIRRFILRRNPGGWMELETYY

>Mizuhopecten yessoensis XP_021367153.1

MASSKVKASQQPRADSYRVAITPGGEDVKKSKKKRKKENLDELKQELEMDEHKIPIEELYERLGSDPNNGLTTERAAMVLARDGPNELTPPPTTPEWVKFCKQLFGGFSMLLWIGAILCFIAYSIQASQYDDPPGDNLYLGIVLTGVVVVTGCFSYYQEAKSSKIMDSFKSMVPQFALVTRSGVKIDTKAENLVVGDVIDVKFGDRVPADVRVISAQGFKVDNSSLTGESEPCSRSADFTNENPLETRNLAFFSTNAVEGICRGIVVKTGDKTVMGRIANLASGLEVGETPIAKEIGHFIHLITGVAVFLGVSFFIIAFILGYFWLDAVIFLIGIIVANVPEGLLATVTVCLTLTAKRMALKNCLVKNLEAVETLGSTSTICSDKTGTLTQNRMTVAHMWFDGHIVEADTSDDQSNATYGAGDETWMSLARVSMLCNRAEFKSGQENVPVLKRECNGDASESALLKCVELSIGDVTGFRHRNKKICEIPFNSSNKYQVSVHETEIPDDPRYLLVMKGAPERILDRCSTVLFNGKDIALDDNFRTAFNAAYMELGGLGERVLGFCDHFLPTDQFQPGYPFDSEEENFPLTGLRFVGLLSMIDPPRAAVPDAVGKCRSAGIKVIMVTGDHPITAKAIAKGVGIISDGSKTVEDIAAERGISVEDVDPRDARAAVIHGADLRDMTPAQIDEILRDHPEIVFARTSPQQKLIIVEGCQRQGQIVAVTGDGVNDSPALKKADIGVAMGIAGSDVSKQAADMILLDDNFASIVTGVEEGRLIFDNLKKSIAYTLTSNIPEISPFLFFILLDIPLPLGTITILCIDLGTDMVPAISLAYEGPESDIMKRQPRDPVKDKLVNERLISMAYGQIGMIQASAGFFVYFVIHSENGFWISKLLGIREQWDAQAVNDLQDSYGQEWTYDQRKTLEYTCHTAFFVSIVVVQWADLIICKTRRLSLFQQGMKNHHLTFGLFFETALAAFLTYCPGLDQGLRMQNLRWTWWFPAMPFSLAIFIYDESRKTIIRRSPGGFVERETYY

>Tridacna squamosa AOR50755.1

MASSKVKTSQQGPRQDSYRYAVTPGDEGGTKKKGKKGKHDMDELKQELTMDEHKIPITELYSRLDTDPNTGLSSEQARIVLEREGPNELTPPKTTPEWLKFCKQLFGGFALLLWIGAILCFIAYSIQASAMENVPGDNLYLGVVLTAVVVVTGCFSYYQEAKSSRIMDSFKNMVPQYGIVIRDGEKLNIKASEMVIGDIVEVKFGDRVPADIRLITGHGFKVDNSSLTGESEPQSRTPEFTNENPLETKNLAFFSTNAVEGTCRGIVIKTGDKTVMGRIANLASGLEVGDTPIAKEIAHFIHLITGVAVFLGVSFFIIAFILGYFWLDAVIFLIGIIVANVPEGLLATVTVCLTLTAKRMAKKNCLVKNLEAVETLGSTSTICSDKTGTLTQNRMTVAHMWFDGRIVEADTSDDQSNATYSKSDPTWLALSRCAMLCNRAEFKNDPENLKQPVLKRECNGDASESALLKCVELSIGNVMEFRKRNRKVCEIPFNSSNKYQVSIHETEDPNDPRSLLVMKGAPERILDRCSSFLRDGVEYPMDDNFRMAFNSAYMELGGLGERVLGFCDFILPADEYPPDYKYDPDDVNFPITGLRFIGLMSMIDPPRAAVPDAVGKCRSAGIKVIMVTGDHPITAKAIAKGVGIISEGSKTVEDIAAERGIPPEEIDPHEAKAAVIHGSDLRDMTPFQIDEILKNHAEIVFARTSPQQKLIIVEGCQRQGQIVAVTGDGVNDSPALKKADIGVAMGIAGSDVSKQAADMILLDDNFASIVTGVEEGRLIFDNLKKSIAYTLTSNIPEISPFLMFILLDIPLPLGTITILCIDLGTDMVPAISLAYEQAENDIMKRMPRDPIKDKLVNERLISMAYGQIGMIQASAGFFVYLVIMGENGFWGSRLMGIREQWDSLAVNDVQDSYGQEWTYYQRKKLEYTCHTAFFVSIVVVQWADLIICKTRKLSLFQQGMKNHHLTFGLFFETALAAFMTYCPGLDQGLRMQNLRASWWFPAMPFSLAIFIYDECRKLILRHRPGGWVEKETYY

>Crassostrea virginica XP_022323941.1

MASTKVKSQQQNPRADSYRYAVTPGGEDPKKGKKKKSKKENLDELKQELEMDEHKIPIEELYARLGTDPTMGLTAQRAKEVLERDGPNALTPPPTTPEWVKFCKLLFGGFSLLLWIGAILCFIAYSIQASAYDDPPGDNLYLGIVLTAVVLVTGIFSYYQEAKSSKIMESFKSMVPQFAVVTRGGKILNIKAEELVVGDVIDVKFGDRVPADVRVITAHGFKVDNSSLTGESEPQTRTAEFTNDNPLETRNIAFFSTNAVEGTCRGVVIRCGDTTVMGRIANLASGLEVGETPIAKEIGHFIHIVTGVAVFLGVSFFIIAFILDYFWLDAVIFLIGIIVANVPEGLLATVTVCLTLTAKRMAKKNCLVKNLEAVETLGSTSTICSDKTGTLTQNRMTVAHMWFDGKIVEADTSDDQTNAAYSGADETWMALARISMLCNRAEFTANQEHLPVLKRECNGDASESALLKCVELSIGKVTEFRARNKKICEIPFNSTNKYQVSIHETENPNDPRCILVMKGAPERILERSSTVLMHGKEVPMDDNFRESFNNAYMELGGLGERVLGFCDYFLPADQYPPGYPYDADDANFPLTGLRFVGLMSMIDPPRAAVPDAVGKCRSAGIKVIMVTGDHPITAKAIAKGVGIISEGSKTIEDLANERGVPVEEIQDTSAAKAAVIHGSDLRDMTPAQIDEVLKNHSEIVFARTSPQQKLIIVEGCQRQGAIVAVTGDGVNDSPALKKADIGVAMGIAGSDVSKQAADMILLDDNFASIVTGVEEGRLIFDNLKKSIAYTLTSNIPEISPFLFFILLDIPLPLGTITILCIDLGTDMVPAISMAYEGAESDIMKRQPRDPFKDKLVNERLISMAYGQIGMIQASSGFFVYFVIMGENGFWMTRLLGLREQWDSQAVNDLQDSYGQEWTYNQRKILEYTCHTAFFVSIVVVQWADLIICKTRRLSLFHQGMKNHHMTFGLFFETFLAAFLTYCPGLEKGLRMQNLRWSWWFPAFPFSIAIFIYDESRKYILRRNPGGFVERETYY

**Na^+^/K^+^-ATPase β subunit**

>Haliotis discus hannai MG767305

MADNKLTVKQKFTNFCSFLYNSETGEVLGRSGRNWGEITFFYIIYYICLAGFFAACFTVFNTTLDDQFPRLIHEDSLIRANPGMGFRPMPDLATTLIRYEMANAKSYEPYVKHINDYLNKNHRNASGDLVDCPDGVTRPDESKACIVDLNSTLATCGEDFGFKIGKPCILLKLNKVFDWTPEPFESDSLPSNMPESLQAKYDQTSIWITCEGENPGDNENLGEIEYLPKQGYDLKYYPYKNQKDYLSPLVFIRLANITPNVGMLIECKAWAKNIWHDRQDRQGSIHFEIIID

>Lottia gigantea XP_009050548.1

MDGRDKTFKQKVDDFMKFLWNSEEKKVMGRSGRSWAEIGFFYLIFYACLAGFFAALIAVFYQTIDEKYPKLMGTDSLLKANPGMGYKPKPDIDSTLIRYAKNEPDTYSVFTTELNGFLDMYANQSSHVECNTTELRPNADLPCKVDIAALTQNCSSANKFGFPDGEPCILLKLNKIFDWTPELYDPAALPDSMPQDVKDIYSSENQIWISCEGENPGDSQNMGTPHFFPSQGFSLKFFPYMNQKGYLAPIVFVKFVGVQRNIGLMIECKAWAKNIQHNRLKKEGSVHFELLVD

>Crassostrea virginica XP_022288413.1

MASSASTGVETATTIPPQNPSIRQRVNDFCTFLYNGDEGTVLGRTGKSWAQIGIFYLIYYSCLSAFFAGMMAIFYQTLDWNYPRLQGPDTLLKQNPGLGFRPIPDVQTTLVRFVKADASTYSPYTDHIQAYLEYYENQNLNPQDGGTVADCDSVTGRRPEKDWDKACKFDLTANLGADCIKQQTFGFDDGMPCILLKLNKIFDWQPESFTNDSVPGDIADLWEPYHITVKCEGENPSDVDNIGPIEYYPQGGFHFKYFPFRNQQAYRSPLVMARFIKPHPGVLIMVECKAYARNIKHDRLEKAGMVHFELMVD

>Mizuhopecten yessoensis XP_021377712.1

MASSASTGVTAASVGPQNQTIGQKFRDFGTFMYNSEEHTVLGRTAKSWGQITLFYAIYYACLSGFFIALIAVFYQTLDWNFPKLSGPDSLLRQNPGMGFRPIPDVTTTLVRFVKGDPTSYSPYTDHMEAYLSYYENENQVTDTGVIVDCDAINGRRPESEWDKACRFDLTQNLGADCVKQQAFGFDDGLPCILVKLNRIFDWMPQMYVNGSGSGIPSEIADMYQNYYVTVKCTGENPADQDNIGPITYYPAGGFHYKYFPFRNQQGYRSPLVFVRFERPTPGVLIMVECRAYAANIQYDRLEHAGSVHFELMVD

>Crassostrea gigas XP_011434772.1

MASSASTGVETAATVSPSNPSLRQRVNDFCTFLYNSEEGSVLGRTGKSWAQIGIFYLIYYSCLSAFFAGMMAIFYQTLDWNYPRLQGPDTLLKQNPGIGFRPIPDVQSTLVRFVKADASTYSPYTDHIQAFLEYYENQNLNPQDGGTVADCDSVTGRRPEKDWDKPCRFDLTANLGADCVKQQTFGFDDGMPCILLKLNKIFDWQPESFTNDTVPAEVADLWEPYHVTVKCEGENPADKDNIGPIEYYPQGGFHFKYFPFRNQQAYRSPLVMARFIRPHPGVLVMVQCKAYARNIRHSQLEKAGMVHFELMVD

>Aplysia californica XP_005111261.1

MEKPTLKQRLDSFAKFLWNGETKQLLGRGGRSWAEIGLFYLVYYACLAGFFAATIAVFYQTIDSHHPKLQGDSSLLKGNPGMGFKPMPDIDTTLVRSTRNPELKKHIKAMTKSITDVLEDYEIDTLDNTDCGGIDETRPNAKTSCRFNYTALTKNCNAGNNFGMDDNQPCVLLKLNKIYGWSPQVWDSSEIDKVPKEIRSSYTPDRIWVYCHGENPADEDNIGHGQIEYYPMQGFPLAFYPYQKQQDYRSPLVFVQFRNVTRNLGLMVECKAFAKNIEVDQTDKQGSVHFELLNDP

>Biomphalaria glabrata XP_013079015.1

MDCIDMNAVRKNASDLCTFLWNGETKEFLGRGKKSWLEITVFYIIFFGVLSAFFISTIVVFHKTIDDVEPKLQGDSSLLGGNPGLGFRPMPDFKTTLIRASNDSKTIEAYVNNINKILQKYKNQTNAVDCSSINETRPLARDHCYFNISALTEHCNEENGYGLKDGQPCVLLKLNKVFGWTPNPWTKMEIQTDSRVPNSIKDVYSPNQIWVHCHGENAGDVDNLGHATEINYYPQQGFPVAYYPYLNQENYTSPLVFVQFRNLTKHVGLMVECIALDRNIKVDKKDKEGSIHFELMVDL

>Octopus bimaculoides XP_014782227.1

MASSDVDPVSAPSQVGFRQSLDDFLVFLYNRDTGEVCGRTGKSWALITIFYVIFYGFLAGFFTATIAVFYTTIDAKYPKLQGKSSLLKGSPGLGYRPRPNYESTLIRFTKGDESMKPYVDNIKDFLQQYNASKYDLEYENCNAISGERETNKDKPCLFDPHSLNAPCLAEPDYGYRSGSPCVLLKLNKIYDWIPEPFGNDSVPEDARDNWDSSFITVTCRGERSTDRENIQDINYFPKYGFPIKYFPYLNQKGYHAPIVIVELKVKRGILLMIECRAYAKNIVVDRLERQGAVHFEILVD

>Capitella teleta ELU02204.1

MASKEVEYQQARPKRTGPGGAWDSFVRFLWNPQAKTCMGRTGSSWAKIVTFYLIYYGLLAGFFAGALMIFYQTLDDIEPRRAGMQSILKGNPGMGFRPMPDVESTLVKFKQGVPDNYAEYVEHIQDFLDCESYNTTDPNAVNCDIETPEEGQVCRFDPDKEAGPCTAANTFGYHEGEPCVLLKLNRIYGWEPEPFNNETINEDNDHAKAAKEALGENIHPEFIGITCEGENPGDVDNRGPAKFYPEHGFPMRYFPFLNQKGYQTPFVFVQFKRPTPGVLINIWCKAWAKNIYHHKKDKAGSIHLELLLD

>Homo sapiens NP_000696.1

MAALQEKKTCGQRMEEFQRYCWNPDTGQMLGRTLSRWVWISLYYVAFYVVMTGLFALCLYVLMQTVDPYTPDYQDQLRSPGVTLRPDVYGEKGLEIVYNVSDNRTWADLTQTLHAFLAGYSPAAQEDSINCTSEQYFFQESFRAPNHTKFSCKFTADMLQNCSGLADPNFGFEEGKPCFIIKMNRIVKFLPSNGSAPRVDCAFLDQPRELGQPLQVKYYPPNGTFSLHYFPYYGKKAQPHYSNPLVAAKLLNIPRNAEVAIVCKVMAEHVTFNNPHDPYEGKVEFKLKIEK

>Penaeus monodon ABV65905.1

MENKQESFRRFLWNPDTKTFLGRTGLSWAKIALFYVFFYLFLAGYFAFMMTVFYSTLDTHAYGRPKYTPTDGSLLRHPGVSIRPRTSTEFMFAAIWFDSTNPSSYQGYVESLDSFVEPYKNNQNVHNPRVYCTAGKRPSGVACNFDIDLLGPCASDRAWGFDKLEPCILVKMNKLLNWIPEPYTSKDLPDDAPEELRTHIQNLEGAGKDTSYTWLSCKPVKGDCIIDYYPEPGFSQVYFPFDNNPGYLPPIVAIKISNLTVNQDVHVVCTLWAKNIVREGFKKQLGEVIFVLSPDDYWIS
