# Supplementary figures and images for: Expression of Na+/K+-ATPase Was Affected by Salinity Change in Pacific abalone Haliotis discus hannai
Source: Front Physiol. 2018 Sep 7;9:1244. doi: 10.3389/fphys.2018.01244 (PMC6137147; doi:10.3389/fphys.2018.01244)

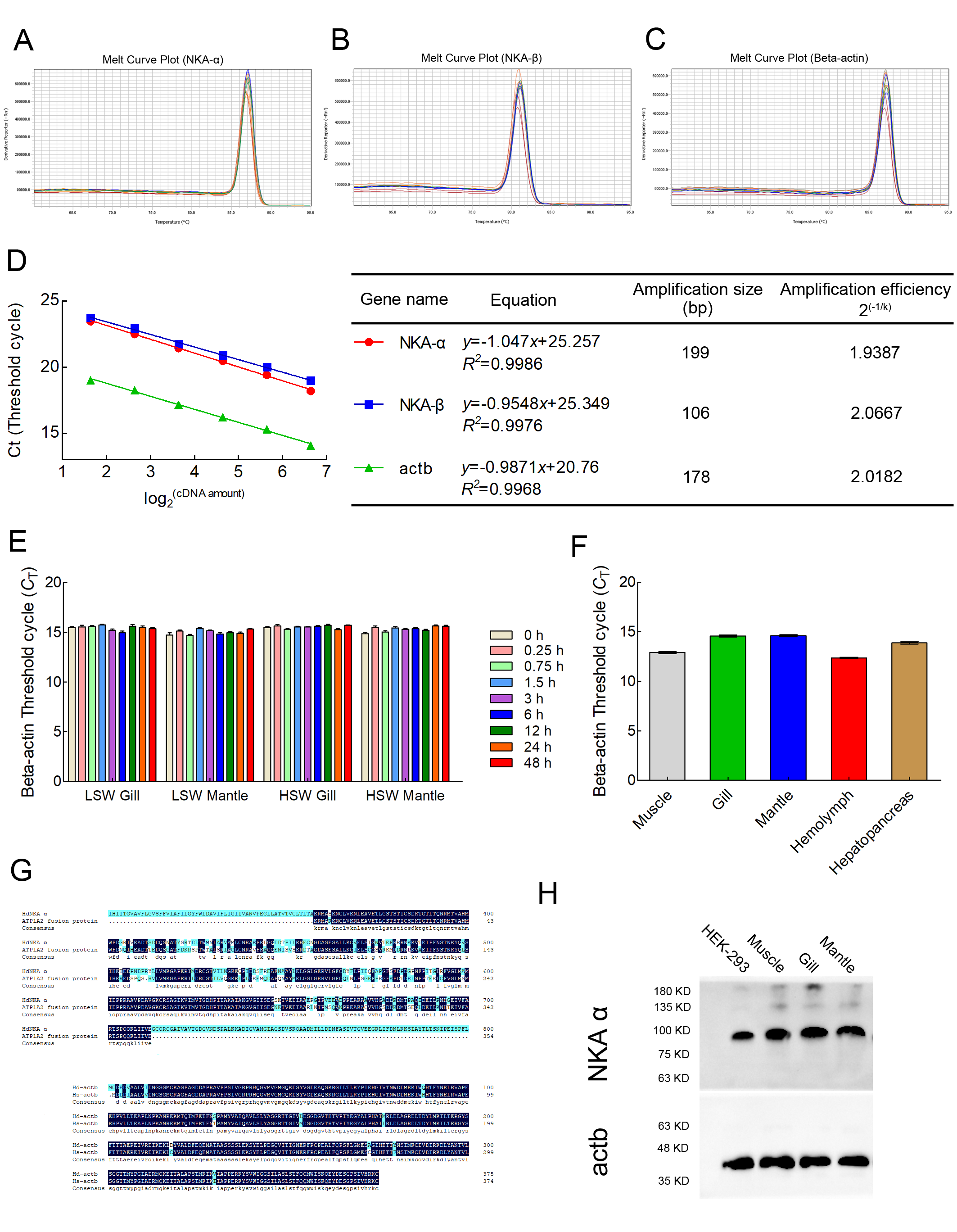

Supplement: Supplementary file 2 [file Image_1.TIF]
